# CTCF binding site classes exhibit distinct evolutionary, genomic, epigenomic and transcriptomic features

**DOI:** 10.1186/gb-2009-10-11-r131

**Published:** 2009-11-18

**Authors:** Kobby Essien, Sebastien Vigneau, Sofia Apreleva, Larry N Singh, Marisa S Bartolomei, Sridhar Hannenhalli

**Affiliations:** 1 Penn Center for Bioinformatics, Department of Genetics, 415 Curie Boulevard, University of Pennsylvania, Philadelphia, PA 19104, USA; 2 Department of Cell and Developmental Biology, 421 Curie Boulevard, University of Pennsylvania, Philadelphia, PA 19104, USA

## Abstract

**Background:**

CTCF (CCCTC-binding factor) is an evolutionarily conserved zinc finger protein involved in diverse functions ranging from negative regulation of MYC, to chromatin insulation of the beta-globin gene cluster, to imprinting of the *Igf2 *locus. The 11 zinc fingers of CTCF are known to differentially contribute to the CTCF-DNA interaction at different binding sites. It is possible that the differences in CTCF-DNA conformation at different binding sites underlie CTCF's functional diversity. If so, the CTCF binding sites may belong to distinct classes, each compatible with a specific functional role.

**Results:**

We have classified approximately 26,000 CTCF binding sites in CD4+ T cells into three classes based on their similarity to the well-characterized CTCF DNA-binding motif. We have comprehensively characterized these three classes of CTCF sites with respect to several evolutionary, genomic, epigenomic, transcriptomic and functional features. We find that the low-occupancy sites tend to be cell type specific. Furthermore, while the high-occupancy sites associate with repressive histone marks and greater gene co-expression within a CTCF-flanked block, the low-occupancy sites associate with active histone marks and higher gene expression. We found that the low-occupancy sites have greater conservation in their flanking regions compared to high-occupancy sites. Interestingly, based on a novel class-conservation metric, we observed that human low-occupancy sites tend to be conserved as low-occupancy sites in mouse (and vice versa) more frequently than expected.

**Conclusions:**

Our work reveals several key differences among CTCF occupancy-based classes and suggests a critical, yet distinct functional role played by low-occupancy sites.

## Abstract 2

CTCF DNA binding sites are classified into distinct functional classes, with distinct biological properties, shedding light on the differing functional roles of CTCF binding.

## Background

CTCF (CCCTF-binding factor) is an evolutionarily conserved, 11 zinc finger protein involved in a wide variety of functions [1]. CTCF is essential for viability, as deletion of the mouse *Ctcf *gene results in early embryonic lethality [2-4]. CTCF was initially discovered as a negative regulator of the *Myc *gene in birds and mammals [5,6], although the function of CTCF as a MYC repressor has recently been challenged [7,8]. CTCF is now also known to serve as a transcriptional activator at various loci [9-13]. In addition, CTCF can also act as an insulator (as chromatin boundary or enhancer blocker), promote intra- or inter-chromosomal interactions, regulate nuclear localization, or participate in the control of imprinting (reviewed in [1,14]). Given the diverse roles of CTCF, it is possible that it binds to a wide variety of DNA motifs, mediated by differential contributions of various zinc fingers [1,6], each facilitating specific protein-protein interactions. Previous investigations for other DNA binding proteins support this possibility. For instance, it was recently shown that, for the glucocorticoid receptor (GR), differences as small as one nucleotide base among the endogenous GR binding sites can have a dramatic impact on GR conformation and activity [15]. Thus, binding sites play an important role in determining function. Using genome-wide location analysis, a majority of the Neuron-restrictive silencing factor (NRSF/REST) binding sites were found to be cell type-specific. Moreover, relative to the ubiquitously bound sites, the cell-type restricted binding sites exhibited a weaker match to the REST consensus binding motif, a greater expression of the neighboring genes, and a greater density of active histone marks in their vicinity [16]. This result suggests existence of distinct functional classes of REST sites. A similar finding has been reported for FOXA2 based on computational analysis of genome-wide location data in mouse liver [17]. As well, a computational analysis of genome-wide transcription factor binding data in yeast concluded that the so-called low-occupancy binding sites are likely to play specific functional roles distinct from the high-occupancy sites [18]. Moreover, for a majority of vertebrate transcription factors, the known binding sites can be statistically partitioned into multiple classes and a predictive model of binding based on multiple classes is often more accurate than a single-motif model [19]. These previous findings motivate a search for functionally distinct classes of binding sites, especially for multi-functional DNA binding proteins, such as CTCF.

Investigation of CTCF binding sites has recently gained momentum [20] owing to advances in ChIP-seq technology, which combines chromatin immunoprecipitation (ChIP) of a protein with high-throughput sequencing of the retrieved genomic sequences and mapping these sequences to the reference genome [21-23]. These large datasets have provided new insights into CTCF biology. For instance, Fu *et al*. [24] found that CTCF sites are flanked by a highly regular array of nucleosomes. Also, a minority of CTCF sites, mostly cell type-specific, tend to demarcate active and repressive domains in the genome [25]. However, most CTCF binding sites are invariant between cell types [22]. The diverse roles played by CTCF, despite its seemingly constitutive binding, are likely facilitated by CTCF's interactions with other proteins such as Cohesins and YY1 [26]. Moreover, it is possible that these diverse interactions are, in turn, facilitated by subtle yet distinct classes of CTCF binding sites. The availability of large CTCF binding site datasets allows us to investigate the existence of functionally distinct classes of CTCF binding sites.

We classified the approximately 26,000 CTCF binding sites in human CD4+ T [21], HeLa, Jurkat [25] and IMR90 [22] cell lines into three classes based on the degree to which they match the known CTCF DNA binding motif, that is, based on their CTCF motif scores. We found a number of significant differences between these classes of CTCF sites in terms of their genomic, epigenomic, transcriptomic, functional and evolutionary properties. Most notably, we discovered that low-occupancy sites are more likely to be specific to a cell type; that low-occupancy sites are evolutionarily more conserved in their flanking regions; that there are significantly fewer than expected transitions between low-occupancy and higher-occupancy classes during human-mouse evolution; that low-occupancy sites are frequently associated with active histone marks, while high-occupancy sites tend to associate with repressive histone marks; that genes in the vicinity of low-occupancy sites have a greater expression in CD4+ T cells relative to the genes near high-occupancy sites; and that genes located between two high-occupancy sites tend to be co-regulated in CD4+ T cells. Thus, our work reveals several key differences among CTCF occupancy-based classes. These differences suggest that the low-occupancy sites are likely to play functional roles distinct from the high-occupancy sites.

## Results

### CTCF sites partition into three occupancy classes

We extracted the ± 100 bp flanking 26,814 human CTCF sites identified in [27] based on genome-wide ChIP-seq data from human CD4+ T cells [21]. We used this sequence window because a large majority of *in vivo *binding sites were found to have a CTCF motif within ± 50 bp [21]. We obtained the CTCF motif (represented as a positional weight matrix (PWM)) from the Ren laboratory website [28] as originally reported in [22]. We used the PWM_SCAN tool [29] to compute the best match score for the CTCF PWM within each 200 bp sequence surrounding a CTCF site; the PWM score varies between 0 and 1 where 1 indicates a perfect match. For comparison, we also computed the PWM scores near the CTCF sites identified in mouse embryonic stem cells [23]. As a negative control, we randomly scrambled the 200-bp regions surrounding the human CTCF sites while preserving the base composition of the original 200 bp sequences. Similar to human sites, we obtained the best CTCF motif score within each randomized 200-bp sequence.

**Figure 1:**
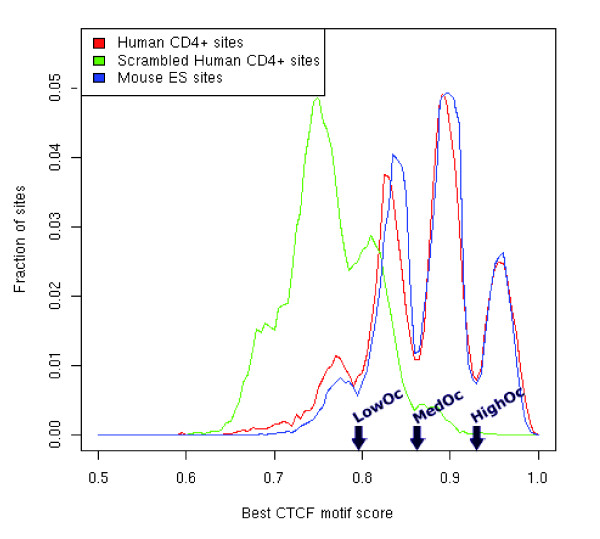
CTCF motif score distribution at the CTCF bound regions in human CD4+ T cells, mouse embryonic stem (ES) cells, and scrambled human sequence. The arrows on the x-axis depict the PWM score ranges assigned to the three classes. For instance, the sites with score between the leftmost and the middle arrow are in LowOc. 'LowOc', 'MedOc', and 'HighOc' classes refer to sites in the first, second and the third mode according to the modal distribution.

**Figure 2:**
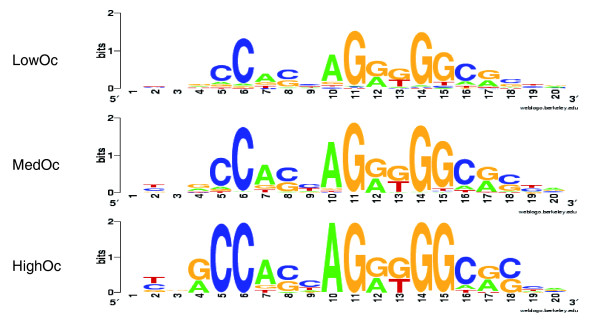
**Motif logos for CTCF sites derived separately from the best scoring sites in each of the three classes**. Top to bottom are LowOc, MedOc and HighOc sites.

As shown in Figure 1, the PWM scores near the CTCF sites form a multi-modal distribution, with a large majority (91%) having a score above 0.79. The origins of this modal distribution are discussed in detail in the Additional data file 1. In the following, we simply use the modal distribution as a guide for partitioning the CTCF binding sites into three score-based classes: low scoring sites (scoring between 0.79 and 0.865) correspond to the first mode, sites scoring between 0.865 and 0.925 correspond to the second mode, and the high-scoring sites correspond to the third mode. These three classes together include 23,891 sites. To verify if the motif score reflects the ChIP enrichment [30], we also analyzed the ChIP-seq tag counts at CTCF binding sites. We found a significant correlation (Spearman rank correlation = 0.28; *P*-value ~ 0) between the PWM score and CTCF Chip-seq tag counts provided in [21]. In particular, tag counts for sites in the third mode are greater than those in the second mode (Wilcoxon test *P*-value ~ E-54), which in turn are greater than those in the first mode (*P*-value ~ E-135). While our site classification is based strictly on the PWM scores, because of their strong correlation with the ChIP-seq tag counts we will, for simplicity, refer to the classes as 'occupancy'-based classes; sites in the first, second and the third mode will be referred to as 'LowOc', 'MedOc', and 'HighOc' classes (see Figure 1). Figure 2 shows the motifs derived separately from each of the three classes. There is a monotonic increase in motif specificity from LowOc to HighOc classes.

For various analyses below, we used as a negative control the 6,432 unoccupied CTCF sites (U class) determined by Kim *et al*. [22], corresponding to genomic locations that strongly match the CTCF motif but were not bound by CTCF either in IMR90 cells [22] or in CD4+ T cells [21]. Due to the specific motif match threshold employed by Kim and colleagues, the vast majority (88%) of unoccupied sites correspond to the MedOc class.

### Low-occupancy sites tend to be cell type-specific

**Figure 3:**
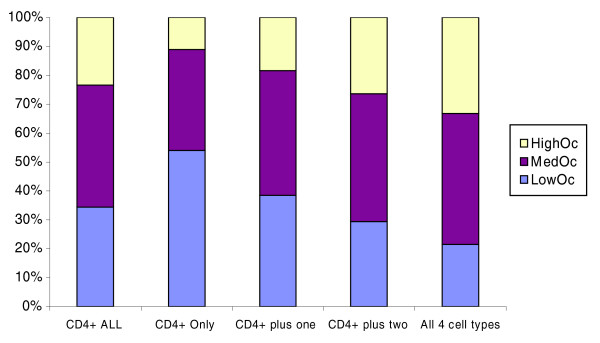
Breakdown of the three CTCF score-based classes for sites either unique to CD4+ T cells or shared with one or more other cell types. The three additional human cell types considered are HeLa, Jurkat, and IMR90. For instance, if a CTCF site is occupied in CD4+ T cells and exactly one of the other three cell types, it is included in the 'CD4+ plus one' category. The bar titled 'CD4+ ALL' refers to all CTCF sites identified in the CD4+ T cells.

In addition to human CD4+ T cells, genome-wide CTCF sites have been characterized in HeLa (19,308 sites) and Jurkat cells (19,572 sites) [25], and in IMR90 cells (13,740 sites) [22]. For each CTCF site identified in the CD4+ T cell, we determined if it was also identified in the other three cell types. A CD4+ T cell CTCF site was deemed to be bound in another cell type if the identified genomic locations in the two cell types were within 200 bp of each other. Figure 3 shows the distribution of CTCF sites into the three occupancy classes, for all CD4+ T cell sites, for sites unique to CD4+ T cells (not identified in any other cell type), and for sites identified in specific numbers of additional cell types. The low-scoring LowOc sites tend to be cell-type specific whereas the high-scoring HighOc sites tend to be ubiquitously bound by CTCF. Specifically, while 23% of 26,814 CD4+ T cell sites are in the HighOc class, only 11% of the sites that are unique to CD4+ T cells are in HighOc and as much as 33% of the 7,428 sites shared by all four cell types belong to the HighOc class. This result is similar to the recent findings for REST sites [16]. In terms of raw numbers, 7,428 (approximately 31%) of CD4+ T cell sites are common to all cell types tested. These common sites are not likely to contain many false positives. In the following analyses, to specifically investigate the inter-occupancy class differences, unless otherwise specified, we will only use the 7,428 common sites. These include 1,595 LowOc (21.5%), 3,367 MedOc (45.3%) and 2,466 HighOc (33.2%) sites. We have provided the genomic locations of these common sites in BED format as Additional data file 2. We have also repeated the analyses using all CD4+ T cells sites. Our conclusions do not change and, in many cases there is a stronger statistical support. When relevant, both analyses are mentioned in the text.

One possible reason for LowOc sites being cell type-specific is that these sites have a lower tag count and thus the chance of their being detected in any given cell-type is low, which may manifest itself as being cell type-specific. To rule out this possibility, we partitioned all sites into five equal-sized bins according to their tag counts and repeated the above analysis separately in each of the bins. We observed the exact same, statistically significant trend of LowOc sites being more cell type-specific in each of the five bins (not shown). Thus, lower tag counts and the detection thresholds do not entirely explain the above observations.

### Broad genomic features of the three CTCF occupancy-based classes

We compared the base composition, as well as the proximity to repeats and to genes, for the three classes and found some significant differences. Within the 200-bp sequence flanking CTCF sites, all three CTCF classes have significantly greater GC content compared with the control U (unoccupied) class (Wilcoxon test *P*-values ~ 0 in all cases). Among the occupancy classes, HighOc sites are associated with a slightly greater, but significant, GC content than LowOc and MedOc sites (*P*-values = E-09 and E-13, respectively), while LowOc and MedOc sites are not significantly different from each other. However, in terms of CG dinucleotide frequencies, while all classes are distinct from the control U class, there is no significant difference between LowOc and HighOc sites (Figure S1 in Additional data file 1).

Next, we calculated the distances between CTCF sites and closest interspersed repeats and low complexity DNA sequences using the RepeatMasker program. As shown in Figure S2 in Additional data file 1, we found that while all classes were significantly farther from repetitive regions relative to unoccupied U sites (all *P*-values < E-08), LowOc sites were farther from repetitive regions relative to HighOc sites (*P*-value = 0.005). However, we did not measure any significant difference in the association of the three CTCF classes with distinct G-banding regions (obtained from the UCSC browser), despite previous reports showing that G-banding correlates with distinct GC content, CpG island density and enrichment in specific repeats [31].

### CTCF site classes associate differentially with various histone marks

The role of various histone modifications in determining spatio-temporal gene expression patterns is well established. Specifically, dozens of histone marks, both methylations and acetylations, have been mapped on a genome scale in CD4+ T cells [21]. Moreover, several histone marks have been shown to correlate, either positively or negatively, with gene expression levels [21,32]. For instance, H3K27me1 is positively associated with gene expression while H3K27me2 and H3K27me3 are negatively associated with gene expression. Apart from the latter two marks, all other marks investigated in this work are positively associated with gene expression. Therefore, histone mark enrichment can be used primarily to assess the association of CTCF site classes with specific patterns of gene expression.

For the histone marks correlated with gene expression, we tested whether there are differences in the histone mark densities (measured by tag densities from ChIP-seq experiments) among the three classes of CTCF sites. For each CTCF site, we computed the density of a histone mark within ± 500 bp of the CTCF site. We compared each pair of CTCF site classes with regard to their tag densities. Table 1 shows the cases with significant *P*-values. In almost all cases, the activation marks are enriched in LowOc relative to MedOc and HighOc, and the two repressive marks are enriched in HighOc relative to LowOc. This observation suggests a functional difference among CTCF occupancy classes; specifically, LowOc sites are more frequently associated with gene activation or euchromatin, while HighOc sites are more associated with gene repression or heterochromatin. We repeated this analysis using all CD4+ T cell sites. Interestingly, all the *P*-values became much more significant, thus providing stronger support to our conclusions (Table S1 in Additional data file 1). Moreover, our conclusions did not change when, instead of using ± 500 bp flanking sequences, we used ± 5 kb flanks. While CTCF has been previously associated both with gene activation and gene repression, our results suggest that different classes of CTCF binding sites may correspond to these distinct functions.

**Table 1:** Comparison of the density of histone marks surrounding CTCF binding sites in different occupancy classes

	LowOc-MedOc	MedOc-HighOc	LowOc-HighOc	LowOc-U	MedOc-U	HighOc-U
H3K4me1	**0.02**			**0**	**0**	**0**
H3K4me2	**1.39E-08**		**3.14E-08**	**0**	**0**	**0**
H3K4me3	**6.59E-09**		**5.63E-11**	**0**	**0**	**0**
H3K27me1	**0.03**		**7.06E-03**	**0**	**0**	**0**
H3K27me2	*1.40E-03*		*0.02*	*5.88E-10*	*3.92E-06*	*3.98E-07*
H3K27me3	*0.04*		*2.23E-03*		0.024	5.28E-04
H3K36me1		**2.79E-04**	**7.08E-05**	**0**	**0**	**0**
H3K36me3						
H3K79me3	**4.10E-05**		**1.90E-06**	**0**	**0**	**0**
H3K9me1	**1.24E-05**		**2.05E-05**	**0**	**0**	**0**
H4K20me1				**0**	**0**	**0**
H2BK5me1				**0**	**0**	**0**
H2AK9ac	**6.49E-03**	**0.05**	**1.47E-04**	**0**	**0**	**0**
H4K12ac	**6.08E-03**		**1.85E-04**	**0**	**0**	**0**
H4K16ac	**3.56E-04**	**0.04**	**2.34E-06**	**0**	**0**	**0**
H2AZ	**1.18E-06**		**1.08E-07**	**0**	**0**	**0**

For each histone mark, the table shows the Wilcoxon one-sided rank-sum test *P*-values for tag density enrichment in one CTCF site class (call this Mi) relative to another class (call this Mj) as well as relative to the control U sites. Column headings represent the CTCF sites classes compared (i.e. Mi~Mj). Only the significant *P*-values are shown. Bold text indicates cases where the tag density for Mi was significantly greater than that for Mj, and italicized text indicates the opposite relation. For instance, in the 'LowOc-MedOc' column, the tag density for H3K27me3 is enriched in MedOc CTCF sites relative to LowOc sites and the null hypothesis was rejected with a *P*-value of 0.04.

The CTCF binding site motif is asymmetric and certain CTCF sites are known to exhibit orientation-dependent activities [33,34]. We define the upstream and downstream of a CTCF site with respect to the CTCF binding motif. Next, we checked whether relative to the orientation of the CTCF motif match there is an upstream versus downstream bias in the histone tag density. For each CTCF site, we tested using the Fisher's exact test whether the partition of all tags between the 5 kb upstream and the 5 kb downstream significantly deviated from expectation, that is, there was an equal split. For this analysis we used only the 5-kb flanking regions as the ± 500 bp flanks did not provide sufficient data for the statistical test. We quantified the overall deviation from expectation by computing, within each class, the fraction of sites for which the number of tags in the upstream 5 kb and the downstream 5 kb significantly deviated from the expected equal split (Fisher's exact test *P*-value ≥ 0.05). By chance alone, we expect approximately 5% of the sites to yield significant deviation from equal distribution. As shown in Table S2 in Additional data file 1, for almost all activating marks but not repressive marks, a large fraction of sites (much greater than 5%) deviates from that expectation. This fraction was consistently (with very few exceptions) greater for LowOc sites than for HighOc sites. Not only is there a general upstream versus downstream tag bias, we found that, in particular, the tag density is higher downstream for most of the activating marks, and the opposite is true for the repressive mark H3K27me3 in the MedOc class. These findings are reported in Table S3 in Additional data file 1 and discussed below.

A direct comparison of the differential tag density bias between different CTCF classes is confounded because, as mentioned earlier, the three classes have different tag densities, which must be controlled for. To do so, while comparing, say, LowOc and MedOc sites, for each site in the LowOc class chosen in random order, we picked a site in MedOc with identical overall tag count in the ± 5-kb flanking region. Each site is selected at most once. This procedure ensures that the selected sites in LowOc and MedOc have identical distributions of overall tag count, thus eliminating the bias. We then compared using Wilcoxon one-sided tests the two classes of sites with respect to their 'tag-density-differential' defined as , where *D*^*u *^and *D*^*d *^represent the upstream and downstream tag densities, respectively. As shown in Table S4 in Additional data file 1, for many activating marks and for the repressive mark H3K27me3, LowOc sites tend to have a greater tag-density-differential relative to HighOc sites. However, we note that even though the above procedure controls for the overall tag count difference between classes, it unavoidably excludes many LowOc sites with high tag density and HighOc sites with low tag densities.

### Genes flanked by high-occupancy CTCF sites have similar expression

Next, we investigated the inter-class differences with respect to the role of CTCF as an insulator. We defined a CTCF block as a genomic region flanked by two consecutive CTCF sites and whose length is at least 50 kb and, at most, 1 Mb. A LowOc-LowOc block corresponds to regions flanked by two LowOc class CTCF sites. MedOc-MedOc and HighOc-HighOc blocks are defined accordingly. We thus derived 168 LowOc-LowOc blocks, 732 MedOc-MedOc blocks and 386 HighOc-HighOc blocks. For each gene pair within a block, we computed their normalized difference in gene expression as *|E1 - E2|/*(*E1 + E2*), where *E1 *and *E2 *are the expression of the two genes in CD4+ T cells, obtained from Novartis GeneAtlas [35]. We denote this quantity as *ΔE*. This method yielded 122 gene pairs in LowOc-LowOc blocks, 975 pairs in MedOc-MedOc blocks and 287 pairs in HighOc-HighOc blocks.

The similarity in expression for adjacent genes may be simply due to genomic proximity. To control for this possibility, for each CTCF block, we also computed the pair-wise gene expression differences in control blocks of the same size that flanked the CTCF block. The flanking control blocks yielded 4,444 pair-wise gene expression differences. We found that the *ΔE *values within LowOc-LowOc, MedOc-MedOc and the flanking control blocks were statistically indistinguishable. However, the *ΔE *values within HighOc-HighOc blocks were significantly smaller relative to LowOc-LowOc blocks (Wilcoxon test *P*-value = 0.03), relative to MedOc-MedOc blocks (*P*-value = 0.005), and relative to the flanking control blocks (*P*-value = 0.004). The distributions of *ΔE *are shown in Figure S3 in Additional data file 1. When we repeated this analysis for sites bound by CTCF only in the CD4+ T cells, the results were more significant. The *ΔE *values within HighOc-HighOc blocks were significantly smaller relative to LowOc-LowOc blocks (Wilcoxon test *P*-value = 0.0001), relative to MedOc-MedOc blocks (*P*-value = 0.0002), and relative to the flanking control blocks (*P*-value = 2.3E-06). We have done additional analyses (data not shown) to ascertain that the above observation cannot be explained by systematically elevated or repressed gene expression within the HighOc-HighOc blocks relative to the corresponding flanks.

### Low-occupancy CTCF sites are associated with gene promoters, with high expression of proximal genes and with greater expression differentials at divergent promoters

As noted above, LowOc sites are associated with a higher density of activating histone marks. To further test whether the LowOc sites are associated with higher gene expression, we examined the expression level in CD4+ T cells of the closest gene to each CTCF site, based on the Novartis GeneAtlas [36]. Figure S4a in Additional data file 1 shows the gene expression values of genes closest to each CTCF site for the different site classes, as well as for the control unoccupied (U) class. For all three CTCF site classes, this expression is higher than for the control U class, with Wilcoxon *P*-values of 6.9E-07 for LowOc-U, 2.0E-07 for MedOc-U and 0.001 for HighOc-U comparisons. Moreover, genes near LowOc sites are expressed at significantly higher levels than genes near HighOc sites (*P*-value = 0.02). We repeated this analysis using all approximately 26,000 CTCF sites in CD4+ T cells [27] and observed the same trend, with more significant differences (Figure S4b in Additional data file 1). The genes near all three CTCF classes show greater expression relative to the U class with Wilcoxon *P*-values of ~ 0 for LowOc-U, 2.9E-13 for MedOc-U and 1.3E-06 for HighOc-U. Genes near LowOc sites are expressed at significantly higher levels relative to genes near MedOc (*P*-value = 0.006) and HighOc (*P*-value = 3.2E-07) sites, while genes near MedOc sites had greater expression than those near HighOc sites (*P*-value = 0.01). These differences remain significant if we only consider genes within 2.5 kb of the CTCF binding site (data not shown). Given the association of LowOc sites with activating histone marks, as well as with higher gene expression, we further hypothesized that LowOc sites should exhibit greater association with promoters. We found that while, overall, only 1,063 (14%) of CTCF sites are within 2,500 bp of a transcription start site (TSS), the LowOc class is significantly enriched among these proximal sites. While LowOc sites represent 21.5% of all CTCF sites, they represent 31.5% of the proximal sites (Fisher's exact test *P*-value = 2.6E-08).

Genes that flank a divergent promoter harboring a CTCF site were previously shown to have a greater difference in expression, relative to the divergent promoters that do not have a CTCF site [37]. We investigated inter-class differences with respect to this property of CTCF sites. We only considered the divergent promoters where the closest gene in either direction was within 100 kb of the CTCF site. This yielded only 37 promoters having a LowOc site, 39 having a MedOc site, 51 having a HighOc site, and 39 having a U site. We measured differential expression as |*E1 *- *E2*|/(*E1 *+ *E2*) where *E1 *and *E2 *are the normalized CD4+ T cell expression of the two genes flanking a divergent promoter. Consistent with the finding in [37], we found that, relative to the divergent promoters containing a U site, the expression differential was greater for promoters containing a CTCF site for all three classes (Mann-Whitney U test *P*-values = 0.002, 0.047 and 0.05 for LowOc, MedOc and HighOc sites, respectively). A direct comparison between the three classes revealed that this differential was greater for LowOc sites relative to MedOc sites (*P*-value = 0.04) and relative to HighOc sites (*P*-value = 0.05). However, all our *P*-values are marginal, perhaps due to very small numbers of divergent promoters containing a CTCF site.

### Low-occupancy CTCF sites are associated with genes down-regulated in CTCF depleted mouse oocytes

In a previous investigation of gene expression changes in mouse oocytes depleted for CTCF by RNA interference, Wan and colleagues [3] observed that a larger fraction of the differentially expressed genes were down-regulated than up-regulated. They also found that, relative to the up-regulated genes, the down-regulated genes were more likely to have a proximal CTCF site, especially in the upstream region of the gene. Here, we further investigate whether there is a biased representation of the three CTCF sites classes near the differentially expressed genes and, specifically, upstream of these genes, using the compilation of CTCF binding sites reported in [3]. As for human CTCF sites, we classified the mouse CTCF sites into LowOc (7,184 sites), MedOc (16,747 sites) and HighOc (12,876 sites) classes. As shown in Figure S5 in Additional data file 1, we observed a significant enrichment of LowOc sites within 10 kb upstream of the down-regulated genes (Fisher's exact test *P*-value = 0.04), further supporting the participation of LowOc sites in gene activation. As a control, we note that LowOc sites are depleted within 10 kb upstream of the up-regulated genes, although this depletion is not statistically significant.

### Distinct DNA motifs and gene functions are enriched near different CTCF site classes

Using a non-redundant set of 235 PWMs corresponding to vertebrate transcription factors from the TRANSFAC database [38], we tested whether transcription factor binding motifs were enriched in the 200 bp flanking the CTCF sites relative to the unoccupied sites. Table S5 in Additional data file 1 shows the 58 motifs that were significantly enriched (false discovery rate ≤ 10%). To identify motifs specifically enriched in each of the three CTCF classes, we used the other two classes as the background control. We found that only one motif corresponding to the transcription factor POU6F1 was enriched in LowOc relative to MedOc and HighOc combined, while no motif was enriched in MedOc relative to LowOc and HighOc combined. However, 16 motifs were enriched in HighOc relative to LowOc and MedOc combined (Table 2). These include the well-known CTCF co-factor YY1 [12].

**Table 2:** TRANSFAC motifs enriched near HighOc sites relative to LowOc and MedOc sites combined

Transcription factor	TRANSFAC PWM ID	Fold enrichment	Fisher *P*-value	FDR
COUPTF	M01036	1.57	0	0
HES1	M01009	1.48	0	0
AP-2gamma	M00470	1.35	0	0
LXR	M00647	1.31	0	0
AP-2	M00915	1.27	0	0
HIC1	M01073	1.25	1.00E-06	3.75E-05
LRF	M01100	1.19	0.000128	0.00335
CACCC-binding_factor	M00721	1.26	0.000133	0.00335
HIC1	M01072	1.19	0.000134	0.00335
GCM	M00634	1.36	0.000231	0.005198
Sp1	M00931	1.24	0.000303	0.006198
CBF_(core_binding_factor)	M01080	1.22	0.000868	0.016275
AP-2alphaA	M01045	1.19	0.001479	0.025598
ZID	M00085	1.25	0.002197	0.035309
YY1	M00069	1.19	0.004876	0.07314
ZF5	M00716	1.11	0.006835	0.096117

Only the motifs with a false discovery rate (FDR) ≤ 10% are shown.

Next, we performed a functional enrichment analysis for the closest gene to each CTCF site, based on Gene Ontology (GO) biological process terms using the DAVID tool [39]. To specifically detect inter-class differences, we used all genes closest to any CTCF site as the background control, and relative to this control we determined the functional enrichment within each CTCF class. As shown in Table S6 in Additional data file 1, the genes near LowOc sites are enriched for several metabolic processes, while the genes near HighOc sites are enriched for neuronal development and differentiation.

We tested whether the genes belonging to functional categories enriched in specific CTCF site classes have unusually high or low expression in CD4+ T cells. For each CTCF class and for each enriched functional GO category, we extracted the closest gene to each CTCF site and annotated it with the corresponding GO term. For each of the subsets of genes obtained, we tested, using the Wilcoxon one-sided test, whether gene expression was higher or lower than for all genes in CD4+ T cells. Certain sets of genes were identical between several functional classes, so we excluded the redundant functional classes from the analysis. As shown in Table S7 in Additional data file 1, for each of the seven functional categories enriched in the HighOc class, the median expression of genes from this category and closest to HighOc sites (we call this gene set the foreground) is lower than the median expression of all other genes (the background control), although this difference is significant in only one case (neuron development). On the other hand, in 7 of the 12 functional categories enriched in the LowOc class, the median expression of the foreground genes is greater than that for the background genes, and this difference is significant in 2 cases, for which the largest number of genes with expression data were available.

### Low occupancy CTCF sites tend to cluster

**Figure 4:**
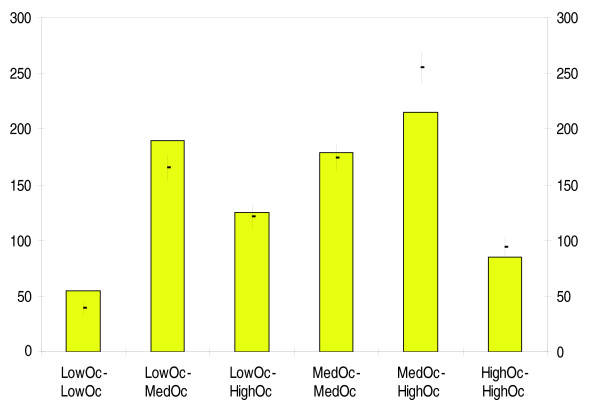
The genomic clustering of CTCF binding sites in various occupancy classes. The bars represent the number of times sites from a specific pair of classes (x-axis label) tend to be adjacent within 1,000 bp on the genome. Mean and standard deviation based on 10,000 randomly permuted data are shown as lines.

We sorted all CTCF sites (from all three occupancy classes) by their genomic location. For each adjacent pair of sites in this sorted list, we noted the classes of the two sites (for example, LowOc and MedOc), and increased by 1 the 'adjacency count' for this class pair (LowOc-MedOc) if the genomic location of the two sites were closer than 1 kb. We thus produced an overall adjacency count for six combinations of classes (LowOc-LowOc, MedOc-MedOc- HighOc-HighOc, LowOc-MedOc- LowOc-HighOc, MedOc-HighOc), indicating the frequency with which the sites in any two classes are adjacent on the genome within 1 kb. We then compared these adjacency counts to a random background, obtained by randomly permuting the class labels while preserving the genomic locations, as well as the total site count for each class. As shown in Figure 4, in real data, LowOc sites tend to be adjacent to each other much more often (*P*-value = 0.008, based on 10,000 permutations) than in the randomized data. LowOc and MedOc sites also tend to be adjacent to each other. No other class combination showed enriched adjacency. This conclusion does not change when we use all CD4+ T cell CTCF sites (data not shown).

### Evolutionary conservation of CTCF classes

**Figure 5:**
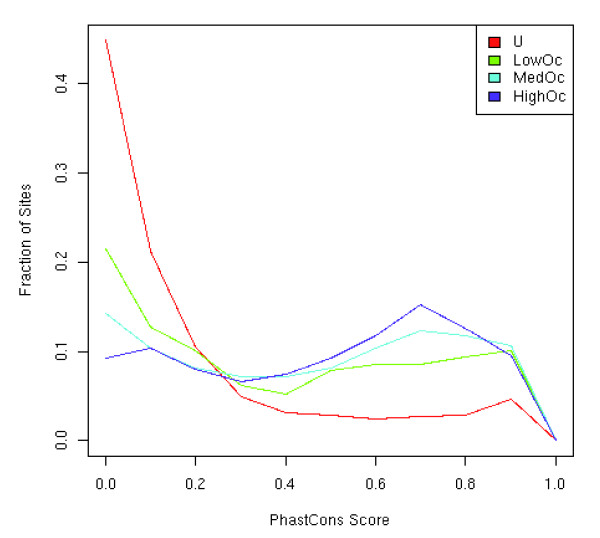
Distribution of the evolutionary conservation for the CTCF sites in the three classes and for the control unoccupied sites. Conservation was measured using the 17-mammal PhastCons score.

For each CTCF site, we extracted the PhastCons cross-species conservation score, based on 17 mammalian species, using the Galaxy web resource [40]. We followed the same procedure for the 50 bp flanking the CTCF sites in either direction. As shown in Figure 5, we found that CTCF sites from each of three occupancy classes are significantly more conserved than U sites (all Wilcoxon *P*-value ~ 0), and HighOc sites are more conserved than MedOc sites (*P*-value = 0.0002), which are more conserved than LowOc sites (*P*-value = 1.2E-12).

However, we found that the genomic sequences flanking LowOc sites are marginally more conserved than those flanking MedOc sites (*P*-value = 0.01), which in turn are more conserved than those flanking HighOc sites (*P*-value = 0.0001); genomic sequences flanking HighOc flanks are not distinguishable in terms of conservation from the those flanking U sites. A greater conservation for lower-occupancy sites in the flanking region is consistent with findings for REST binding sites [16], although the authors considered larger flanking regions.

**Figure 6:**
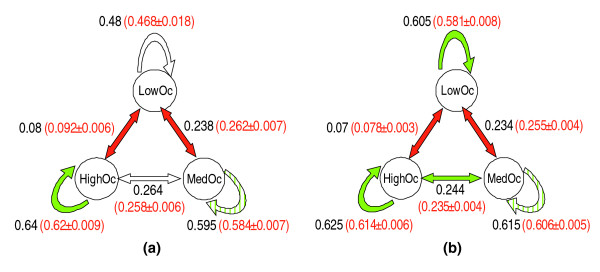
Human-mouse conservation of occupancy classes and transitions among the classes. The arrows represent transitions between human and mouse at the aligned CTCF site locations. The numbers next to arrows represent the fraction of times that transition was observed in the real data; those in parentheses show the mean and standard deviation of this transition probability based on 1,000 simulations, according to a stringent evolutionary model. Green colored arrows indicate that the observed transition probability was significantly greater (simulation based *P*-value ≤ 0.05) than expected and red colored arrows indicate that the observed transition probability was significantly smaller than expected. The light green shaded arrows indicate near-significance (0.05 <*P*-value ≤ 0.07). **(a) **Based on the CTCF sites shared among the four cell types. **(b) **Based on all CTCF sites in CD4+ cells.

Next, using 3,930 orthologous CTCF sites (656 LowOc, 1,814 MedOc, 1,460 HighOc) that were aligned without gaps between human and mouse, we investigated the conservation of CTCF classes between the two species by testing whether CTCF sites belonging to a specific class in human tend to belong to the same class in mouse, and vice versa. More specifically, we estimated for each pair of classes *i and j*, where *i*, *j *∈ {*LowOc*, *MedOc*, *HighOc*}, the probability that a class *i *site in one species (human or mouse) corresponds to a class *j *site in the other species. The resulting 3×3 matrix of probabilities is referred to as the 'class-transition probability matrix' (CTPM). We compared the CTPM estimated from the real data with a control CTPM calculated from datasets wherein each site in one species (coming from the real genomic sequence) had been mutated according to a very stringent evolutionary model to generate the corresponding orthologous site in the other species (see Materials and methods; Additional data file 1). We performed 1,000 such simulations, 500 by fixing the human sites and generating synthetic orthologous mouse sites and 500 by fixing the mouse sites and generating synthetic orthologous human sites. Assuming a reversible Markov process of evolution, our simulation captures overall mutations along the two branches connecting mouse and human from the common ancestor, and does not imply a directionality of evolution from human to mouse or vice versa. We compared the *CTPM *[*i*, *j*] for the real datasets to the *CTPM *[*i*, *j*] calculated from the 1,000 random sets. As shown in Figure 6a, we found that all classes are conserved more often than expected by random. In addition, compared to expectation, the LowOc-MedOc and LowOc-HighOc transitions are significantly rarer, while the MedOc-HighOc transitions are more common (although not significant). When we repeated this analysis with all 9,903 CD4+ T cell sites (2,473 LowOc, 4,559 MedOc and 2,871 HighOc) orthologous between human and mouse, our conclusions found a greater statistical support (Figure 6b).

The above class-conservation analysis is based on an *ad hoc *definition of classes based on the modal distribution of CTCF PWM scores. To exclude any artifact due to the partition, we also tested whether, within the entire range of PWM scores, the score difference between human and mouse is smaller than expected, using the same evolutionary model as above. For any given CTCF site, we computed the difference *Δs *between the PWM scores for human and mouse sequences. We then compared the distribution of *Δs *with the distribution of randomly generated site pairs, as described above. We tested the null hypothesis '*Δs *for real data is less than the *Δs *for randomized data' using the Wilcoxon test. We performed 1,000 Wilcoxon tests for the 1,000 randomly generated set of sites. The null hypothesis was rejected (*P*-value ≤ 0.05) in 75% of the cases while the random expectation is only 5%. When we repeated this analysis with all CD4+ T cell sites, we found that the null hypothesis was rejected (*P*-value ≤ 0.05) in 99.7% of the cases. Thus, the *Δs *values in the real dataset tend to be smaller than expectation based on a stringent evolutionary model.

### The three CTCF binding site classes exhibit disparate word preferences

While we have defined the three classes of CTCF binding sites based on the degree of match to a single PWM, next we investigated whether there are distinct motifs or nucleotide 'words' that occur preferentially in only one of the classes, which may provide the basis for functional differences between the classes. Even though the CTCF binding site is 20 bp long, it has two conserved cores - one spanning from bases 4 to 8 and the other from bases 10 to 18 (Figure 2). We explored the sequence differences between the three classes of sites by examining the *k*-mers (nucleotide sequences *k*-bp long) that occur preferentially in each of these sites. We estimate the enrichment of a *k*-mer in one of the classes - for example, LowOc - relative to the other two classes combined using Fisher's exact test based on the *k*-mer frequencies and total number of sites in different classes. We then correct the enrichment *P*-values for multiple testing and use a false discovery rate threshold of 1% (or 0.01%). In the shorter 5-bp core (*k *= 5) there are 216, 120 and 30 distinct *k*-mers in LowOc, MedOc and HighOc sites, respectively. This reflects less variability in HighOc sites, consistent with its strong match to the consensus (Figure 2). Of these, 15 *k*-mers preferentially occur in the LowOc relative to a background consisting of MedOc and HighOc sites. Likewise 12 *k*-mers preferentially occur in MedOc and 11 in the HighOc sites. As expected, there is little or no overlap in these preferential *k*-mers. In the larger 9-bp core (*k *= 9) there are 610, 354 and 78 distinct *k*-mers in LowOc, MedOc and HighOc sites, respectively. Of these, 5, 3 and 20 preferentially occur in the LowOc, MedOc and HighOc sites, respectively, and there is no overlap between the 3 sets of enriched *k*-mers. Thus, in general, there are several *k*-mers that occur preferentially in one of the CTCF binding site classes. These *k*-mers are reported in Table S8 in Additional data file 1. However, the functional significance of these differences is not immediately clear and can be best assessed experimentally.

## Discussion

### CTCF as a model for investigating DNA binding site classes

It is becoming increasingly clear that for many DNA binding proteins, their binding sites fall into distinct classes [15-19,41]. Especially for multifunctional proteins, the functional consequence of DNA binding may depend on the specific binding site class, as well as on the genomic and epigenomic contexts. CTCF, an 11 zinc finger protein, was first characterized as a transcriptional repressor [5,6]. However, several recent studies have implicated CTCF in the activation of the mouse *H19 *[10] and *Tsix *genes [11,12], as well as the human *HLA-DRB1 *and *HLA-DQA1 *genes [13]. In addition, CTCF can act as an enhancer blocker, participate in chromatin barriers, promote interaction between distant sequences and target localization of bound sequences into specific nuclear compartments [14,25]. Based on these observations, combined with the fact that the combinatorial use of CTCF's 11 zinc fingers may facilitate the ability of CTCF to bind to divergent sequences [6], we conclude that CTCF is a *bona fide *multifunctional binding protein. Recently, CTCF binding sites have been mapped to the human genome in several cell types, thus making CTCF an ideal candidate for an investigation of possible binding site classes and their distinct functional roles. Although it would be desirable to classify CTCF sites based on functional mechanisms, it is currently difficult, not only because of insufficient data, but also because these mechanisms may not be mutually exclusive. For instance, a single CTCF function (for example, in chromatin looping) may affect a variety of transcriptional outputs and may be interpreted as affecting distinct functions depending on the assay. Therefore, we have classified CTCF binding sites based on their similarity to the published PWM for CTCF [22]. Many previous works have suggested a similar classification of the binding sites for other DNA binding proteins [16-18]. However, specific differences in the sequences of the binding sites corresponding to different classes are likely to underlie the functional differences between the binding site classes [15]. Given that 91% of CTCF enriched regions have a binding site score above 0.79, any specific sequence differences between classes are likely to be subtle. This is consistent with recent experimental work in GR binding sites that has established that even single nucleotide differences in binding sites can significantly alter their regulatory activity [15]. In our case of CTCF, these subtle differences are reflected in small but detectable differences in overall motif score. Even though we find that several *k*-mers occur preferentially in each binding site class, further experiments are needed to determine the extent to which these sequence differences underlie the functional differences.

### Differential evolutionary conservation and cell-type specificity among the classes of CTCF sites

The high-occupancy HighOc sites are more conserved than LowOc sites, but the sequences flanking LowOc sites are more conserved than those flanking HighOc sites. The lower conservation of LowOc sites may be related to the observation that they are more often organized in clusters and may thus provide redundant functionality at a given locus. As a possible explanation for the greater conservation of sequences flanking LowOc sites, the function of LowOc sites may rely more on interactions with the local genetic and epigenetic context. Such interactions are likely to be cell type-specific, since the CTCF binding at low-occupancy LowOc sites is more variable between cell types than at HighOc sites; this finding is consistent with a recent study on REST/NRSF binding sites [16]. A greater variability in CTCF binding at the low-occupancy sites is also consistent with the idea that changes in gene expression during cellular differentiation require a rapid clearance of regulatory factors from their binding sites. It is also possible that HighOc sites are highly conserved because of their role in cellular morphology as well as neuronal morphology and differentiation, whereas the functions of LowOc sites may be preserved by virtue of their conserved flanking regions.

In addition, we found that the various classes of CTCF binding sites are evolutionarily conserved - that is, the evolutionary transition of a LowOc site to either a MedOc site or to a HighOc site is less frequent than expected. This observation further supports the idea that the LowOc sites accomplish distinct functions and are not interchangeable with a HighOc site, consistent with findings in [41].

### Low occupancy CTCF sites have a greater association with euchromatic histone marks and higher gene expression

We found that LowOc sites are enriched for euchromatic histone marks and are associated with higher levels of gene expression. Consistently, the expression of genes in the enriched GO categories near HighOc sites in CD4+ T cells is generally lower than background, whereas that of genes in the enriched GO categories near LowOc sites is higher. Moreover, the predominance of a transcriptional activation function for LowOc sites was further supported by analyzing changes in gene expression in mouse oocytes depleted for CTCF. Given that the LowOc class is significantly enriched within 2,500 bp of TSSs compared to other classes, the transcriptional activation could be achieved, at least for these TSS-proximal LowOc sites, by a direct interaction with the transcription machinery leading to the recruitment of RNA polymerase II [42]. However, less than 20% of all CTCF sites are near TSSs; thus, other mechanisms must play a role in LowOc-mediated transcriptional activation.

### Upstream versus downstream bias in histone mark densities flanking CTCF sites

We found that most euchromatic marks were differentially enriched between the two flanks of CTCF sites (Table S3 in Additional data file 1). Moreover, additional analyses (Tables S2 and S4 in Additional data file 1) show that this differential is greater for the LowOc sites. These findings, together with our observed enrichment of LowOc sites at TSSs, are consistent with the previously described differential pattern of certain euchromatic marks relative to TSSs [21]. However, our direct comparison of classes also revealed that the differential for the heterochromatic mark H3K27me3 is significantly higher for LowOc than for HighOc sites (although the *P*-value = 0.04, which is marginally significant). Previous studies have identified a significant association of CTCF binding with the boundaries of repressive chromatin domains marked by H3K27me3 [25], as well as with the boundaries of lamina-associated domains [43]. We also found a greater differential in expression of the genes flanking divergent promoters harboring a LowOc site, similar to the findings in [37] for CTCF binding sites in general. Taken together, these results suggest that there is a marginally higher proportion of LowOc sites at the border of chromatin domains and lamina-associated domains. Since CTCF binding at LowOc sites tends to be more cell type-specific, our observation is also consistent with the previous report that CTCF binding at the border of chromatin domains is mostly cell type-specific [25].

In addition, we observe that the density of most of the euchromatin marks is higher downstream than upstream of CTCF sites (Table S3 in Additional data file 1). This result is consistent with oriented binding of CTCF to DNA [44,45] and orientation-dependent activities of CTCF sites [33,34]. The enrichment of the heterochromatin mark H3K27me3 upstream of MedOc sites is reminiscent of the pattern reported at the border of chromatin domains [25]. This suggests that, in addition to LowOc sites, CTCF binding at MedOc sites could also accomplish a chromatin barrier function. However, the significance of the observed enrichment of the heterochromatin mark H3K27me2 downstream of LowOc and MedOc sites is not clear, and requires further analysis.

Our analyses suggest that HighOc sites tend to act less often than LowOc sites as chromatin barriers but, paradoxically, we also find a greater tendency among HighOc sites for delimiting domains of co-regulated genes. This insulator activity of HighOc sites may thus rely on distinct mechanisms, such as facilitating intra- and inter-chromosomal interactions [46], and may involve additional interacting proteins, such as YY1, whose consensus sites are found to be enriched near HighOc sites.

### Occupancy classes of functionally characterized CTCF sites

We have compiled a list of approximately 150 experimentally characterized CTCF binding sites at well studied gene loci, many of which correspond to imprinted genes (Additional data file 3). We determined the class of CTCF binding site at each of these loci. Strikingly, almost none of the CTCF sites found at imprinted loci, including *H19/Igf2*, *Kcnq1/Kcnq1ot1*, *Dlk1/Gtl2*, *Rasgrf1 *and *Grb10*, belong to the HighOc class. The same was observed for CTCF sites associated with the *Xist*, *Tsix *and *Xite *loci, which regulate X chromosome inactivation. Specifically, *Xist *and *Tsix *expression is imprinted in extraembryonic lineages, and *Xist *expression is monoallelic, but not imprinted, in somatic cells [47]. At most of these monoallelically expressed loci, CTCF sites are known to be organized in clusters, consistent with their low occupancy. Our observation also suggests that other properties of low-occupancy CTCF sites may be important for monoallelic gene expression, likely to allow the differential binding of CTCF at the two alleles. It is tempting to speculate that such properties have evolved from the ability of low-occupancy sites to promote cell-type specific binding of CTCF. Most imprinted loci, as well as X chromosome inactivation, evolved recently in mammals, after the separation of marsupials and monotremes from eutherians [47-51]. Further analysis needs to be done to investigate whether the CTCF class distinction has a parallel evolutionary origin. In contrast, at the *beta-globin *and *olfactory receptor *loci, a majority of CTCF sites belong to the HighOc class. In this case, high occupancy binding of CTCF may be important to ensure specific chromosomal conformation via intra- and inter-chromosomal interactions [52]. CTCF sites at the c-*myc *locus also belong to the HighOc class, consistent with the constitutive binding of CTCF independent of the transcriptional status of c-*myc *[7].

## Conclusions

We have found several statistical genome-wide trends suggestive of differences in functional roles played by CTCF binding sites in different occupancy classes. These trends are summarized in Table S9 in Additional data file 1. Several lines of evidence indicate that CTCF bound to LowOc sites interact with promoters and are likely to be involved in gene activation. These include: greater association of LowOc sites with transcription start sites; greater CD4+ T cell expression of genes closest to a LowOc site; greater association of LowOc sites with euchromatic marks; and greater association of LowOc sites with down-regulated genes in a mouse knock-down study. LowOc sites can also be interpreted as playing a role in establishing chromatin barrier. For instance, there is a greater expression difference between genes that flank a LowOc site in a divergent promoter; however, this could be a side effect of its role as an activator of gene expression. Also, LowOc sites exhibit a greater upstream versus downstream bias for the repressive H3K27me3 mark. In comparison, HighOc can be interpreted as playing a role in gene repression, as well as in establishing gene co-expression domains flanked by CTCF insulator sites. HighOc sites have a greater association with repressive marks and also a lower expression for nearby genes, especially the ones with functions enriched near HighOc sites. A role for HighOc sites as an insulator is supported by a lower expression difference between genes within blocks flanked by HighOc sites. However, it must be noted that the inter-class differences in various properties, while being statistically significant, are often small and do not immediately provide clear biological insight into the potential functions of these proposed classes. Further analyses and experimental work needs to be done in order to propose a refined model that explains the structural underpinnings of the observed differences. Likewise, the causality between CTCF binding and associated features needs to be tested experimentally.

In summary, our study provides a detailed comparative analysis of occupancy-based classes of CTCF sites, based on their genomic, epigenomic, transcriptomic, evolutionary and functional attributes. We believe that a similar study of other DNA binding proteins should elucidate the mechanistic basis underlying their multiple functional roles. A thorough and wider application to additional multifunctional proteins may result in the emergence of general rules.

## Materials and methods

### Motif enrichment near various CTCF classes

We first obtained 546 DNA binding motifs as PWMs corresponding to vertebrate transcription factors from the TRANSFAC database [38]. Many PWMs corresponding to structurally related transcription factors are highly similar and do not provide independent information. Using a relative-entropy based measure of similarity between a pair of PWMs described in [53], we pared down the PWMs to a set of 235 relatively non-redundant PWMs. We tested for the enrichment of these 235 motifs near the three classes of CTCF sites. Given three sets of CTCF sites corresponding to LowOc, MedOc and HighOc classes, for each site location we extracted the ± 100 bp flanking sequence. Using the PWM_SCAN tool [29], we identified all instances of putative binding sites for the 235 PWMs in all 200-bp sequences. We used a *P*-value cutoff of 0.0001 for the PWM match to define the binding sites. For each motif, we compared the number of occurrences in the sequences flanking a specific CTCF class relative to a specific control (as defined in the Results section). We estimated the *P*-value of enrichment using Fisher's exact test. We then estimated the false discovery rate for each *P*-value threshold using the q-value function in the R package [54]. We have reported the motifs with q-value ≤ 10%.

### Evolutionary model to assess the intra-class conservation and inter-class transition

Starting with a set of experimentally determined CTCF sites in human (in LowOc, MedOc, or HighOc classes), and using the genome-wide human-mouse alignment from the UCSC database, we identified a subset of sites that were aligned in mouse without any gaps. When using sites shared among all cell types, this process resulted in 3,930 sites, as opposed to using all CD4+ T cell sites, which resulted in 9,903 aligned sites. For the given set of human-mouse aligned sites, we scored the mouse counterparts of the human sites using the same CTCF PWM and classified them into LowOc, MedOc or HighOc sites. A small fraction (< 5%) of sites was further excluded at this stage as their score was below the LowOc threshold. We computed the CTPM as a symmetric 3×3 matrix where *CTPM *[*i*, *j*] indicates the probability that a site in the class *i *in one of the species (human or mouse) corresponds to a site in class *j *in the other species. Here *i*, *j *∈ {*LowOc*, *MedOc*, *HighOc*}. In other words, a class *i *site corresponds to a class *j *site during evolution. Specifically, let *n1*_*h *_be the number of LowOc sites in human, and *n1*_*m *_be the number of LowOc sites in mouse. *n2*_*h *_and *n2*_*m *_are defined correspondingly. Also, let *n12 *be the number of sites that are LowOc in human and MedOc in mouse, and let *n21 *be the number of sites that are MedOc in human and LowOc in mouse. Therefore, the probability of transition between LowOc and MedOc is estimated as (2 × *n12 *+ 2 × *n21*)/(*n1*_*h *_*+ n1*_*m*_*+ n2*_*h*_*+ n2*_*m*_). The multiplicative factor of 2 in the numerator accounts for the transition from human to mouse and mouse to human. For instance, if we restricted our analysis only to human-to-mouse transitions then the probability would be estimated as (*n12 + n21*)*/*(*n1*_*h *_*+ n2*_*h*_). It is important to note that when we refer to transitions from human to mouse or mouse to human, we are not claiming that one species evolved from the other but rather using a site in one species as a reference of comparison for a site in the other species. We have used both formulae (data not shown) and our conclusions do not change. Here we only present the 'symmetric' case (the first of the two above formulae) for simplicity of exposition.

Next we compare the estimated transition probabilities against random expectation by simulating evolution based on a stringent evolutionary model as follows. We assume that each base in the 20 bps long CTCF sites evolves (including mutation and selection) according to a distinct 4×4 base transition probability matrix. Moreover, we also do not assume that these base transition probabilities are identical in the human to mouse and in the mouse to human directions. We estimated 2 sets of 20-base transition probability matrices by simply counting these transitions in the set of aligned human-mouse sites, regardless of the CTCF class.

Given a CTCF site S in one species, say human, we generate a synthetic mouse site, while applying the following restrictions. First, we only 'mutate' the positions in the site that are different between human and mouse, that is, mutated in the real data. This restriction very strictly controls for the variation in mutation rates across genomes as well as evolutionary rates across different positions within the CTCF sites. Second, given that the hyper-mutability of CG dinucleotides is conservative, we do not mutate CG dinucleotides. Thus, given a human CTCF site, we mutate exactly the positions that are mismatched between human and mouse, according to the species-specific and position-specific base transition probability matrices.

In a given iteration, we fixed the species, say to human, created an entire set of mouse sites as described above and computed the class-transition-probability matrix as described above. We generated 500 sets of aligned sites by fixing the human site and generating the mouse counterpart, and another 500 sets of aligned sites by fixing the mouse site and generating the human counterpart. For each class-transition-probability estimated from the real data, say *CTPM *[*i*, *j*], we have 1,000 probabilities based on our simulations. We estimate the significance of *CTPM *[*i*, *j*] based on the 1,000 simulated probabilities.

## Abbreviations

ChIP: chromatin immunoprecipitation; CTPM: class transition probability matrix; GO: Gene Ontology; GR: glucocorticoid receptor; NRSF/REST: Neuron-restrictive silencing factor; PWM: positional weight matrix; TSS: transcription start site.

## Authors' contributions

SH conceived the study. KE, SA, and SH did the analysis with help from LNS. SV provided many of the datasets for the analysis and provided the literature review for the discussion. SH, MSB, KE, and SV wrote the manuscript.

## Additional data files

The following additional data are available with the online version of this paper: additional discussion of the trimodal distribution of CTCF PWM scores, as well as additional figures and tables supporting results in the paper (Additional data file 1): coordinates of the CTCF sites shared among the four cell types (Additional data file 2); a list of experimentally determined CTCF sites in human and mouse and their occupancy classes (Additional data file 3).

## Supplementary Material

Additional data file 1 Figure S1: distribution of C+G and CG dinucleotide fractions in the ± 100-bp region flanking the three classes of CTCF sites. Figure S2: distributions of distances from a repeat for each of the three CTCF classes. Figure S3: distributions of differences in gene expression for gene pairs within CTCF blocks. Figure S4: expression distribution for the gene closest to a CTCF site for each of the three classes. Figure S5: distributions of mouse CTCF sites in the three classes. Table S1: class versus class comparison of densities of various histone marks in the 500-bp region flanking CTCF sites. Table S2: fraction of sites within each CTCF site class with unequal distributions of a specific histone mark in upstream and downstream regions (± 5 kbp). Table S3: comparison of upstream and downstream tag counts of various histone marks for each class of CTCF site. Table S4: class versus class comparison of tag-density-differential for various histone marks. Table S5: motifs enriched in the 200-bp flanking region of bound CTCF sites. Table S6:functional enrichment near each of the three CTCF site classes. Table S7: tendency of functional categories enriched near CTCF site classes to have higher or lower expression than background genes. Tables S8: k-mers enriched in each of the three classes of sites relative to the other two classes. Table S9: summary of the main observed differences between the three classes of site. Click here for file

Additional data file 2 Coordinates of the CTCF sites shared among the four cell types. Click here for file

Additional data file 3 Experimentally determined CTCF sites in human and mouse and their occupancy classes. Click here for file
