# Supplementary material for: CTCF binding site classes exhibit distinct evolutionary, genomic, epigenomic and transcriptomic features
Source: Genome Biol. 2009 Nov 18;10(11):R131. doi: 10.1186/gb-2009-10-11-r131 (PMC3091324; doi:10.1186/gb-2009-10-11-r131)
Supplement: Additional data file 1 — Figure S1: distribution of C+G and CG dinucleotide fractions in the ± 100-bp region flanking the three classes of CTCF sites. Figure S2: distributions of distances from a repeat for each of the three CTCF classes. Figure S3: distributions of differences in gene expression for gene pairs within CTCF blocks. Figure S4: expression distribution for the gene closest to a CTCF site for each of the three classes. Figure S5: distributions of mouse CTCF sites in the three classes. Table S1: class versus class comparison of densities of various histone marks in the 500-bp region flanking CTCF sites. Table S2: fraction of sites within each CTCF site class with unequal distributions of a specific histone mark in upstream and downstream regions (± 5 kbp). Table S3: comparison of upstream and downstream tag counts of various histone marks for each class of CTCF site. Table S4: class versus class comparison of tag-density-differential for various histone marks. Table S5: motifs enriched in the 200-bp flanking region of bound CTCF sites. Table S6:functional enrichment near each of the three CTCF site classes. Table S7: tendency of functional categories enriched near CTCF site classes to have higher or lower expression than background genes. Tables S8: k-mers enriched in each of the three classes of sites relative to the other two classes. Table S9: summary of the main observed differences between the three classes of site. [file gb-2009-10-11-r131-S1.DOC]

# Supplementary Material

**Trimodality of the CTCF PWM scores**

The CTCF PWM scores for reported CTCF bound sites follow a trimodal distribution both in human CD4+ T cells and in mouse ES cells. However, there is no such modality in the corresponding ChIP-seq sequence tag count, thus the modality is likely a property of the PWM itself and unlikely to represent a trimodal distribution of the actual binding affinity. In the PWM reported by the Ren laboratory, which is based on 229 CTCF sites, only one of the 4 bases is permitted at key positions 5, 6, 10, 11, 14 and 15. Thus a non-consensus base at one or more of these key positions will result in a large decrease in the PWM match score. The modal distribution of the PWM scores is precisely the consequence of discrete shifts in match score. In other words, each mode corresponds to a set of consensus bases and the spread around that mode is caused by smaller changes in score attributable to other non-key positions in the binding site. However, we did not find a correspondence between a mode and a specific combination of nucleotides at key positions. Thus the modes are likely due to multiple combinations of nucleotides, whose precise determination would require further examination. In the current work, we used the modal distribution simply as a guide for partitioning the sites into “occupancy”-classes, without any further claims. Because this partitioning is based solely on the sequence and the same partitioning method is applicable in human and mouse, it allowed us to compare the properties of CTCF sites subclasses between cell-types and species.

**Supplementary Figures and Tables**

| 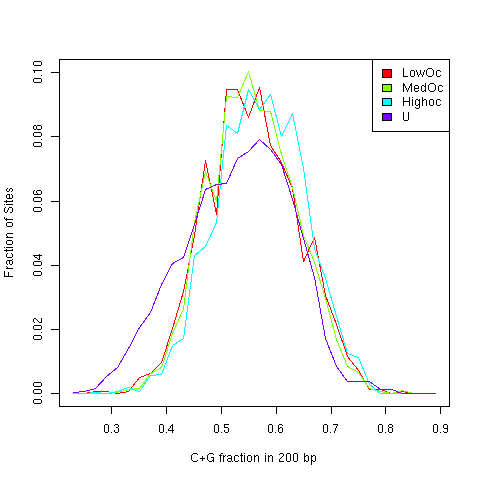 | 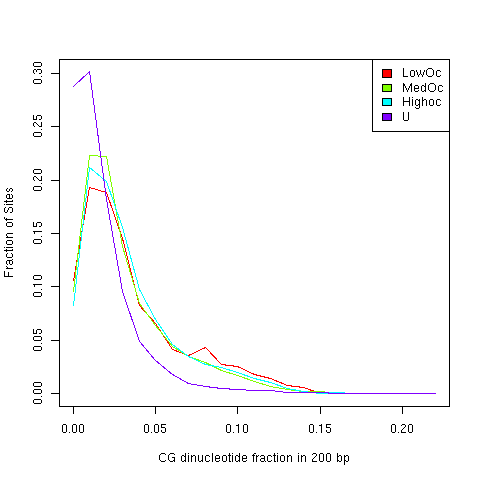 |
| --- | --- |

***Figure S1*.** Distribution in the ±100bp region flanking CTCF sites from the different classes of (a) the C+G fraction (b) the CG dinucleotide fraction.


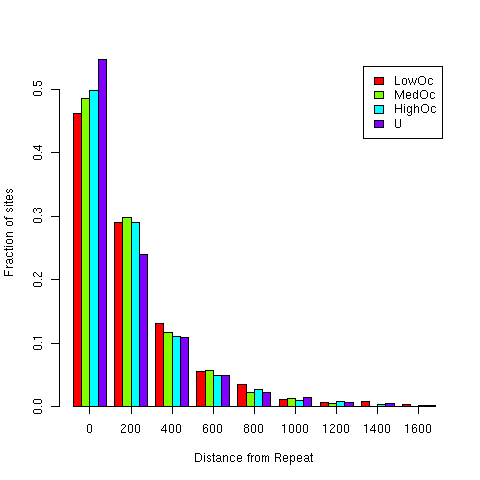


***Figure S2****. Distribution of distances from a repeat region for the different CTCF classes and the control unoccupied class.*


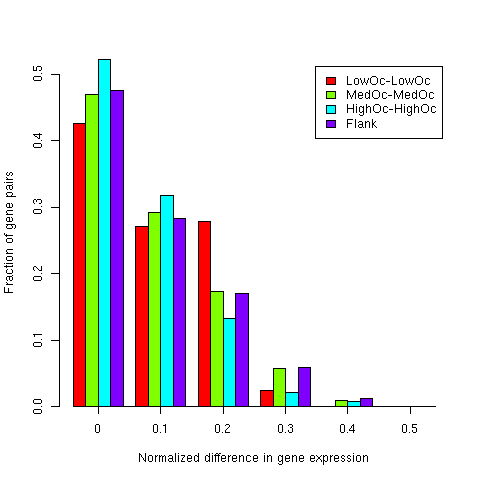


***Figure S3.*** *Distribution of normalized differences in gene expression for pairs of genes within CTCF blocks flanked by CTCF sites belonging to the same class. For comparison the expression differences for gene pairs in size-matched flanking blocks are also shown.*

| 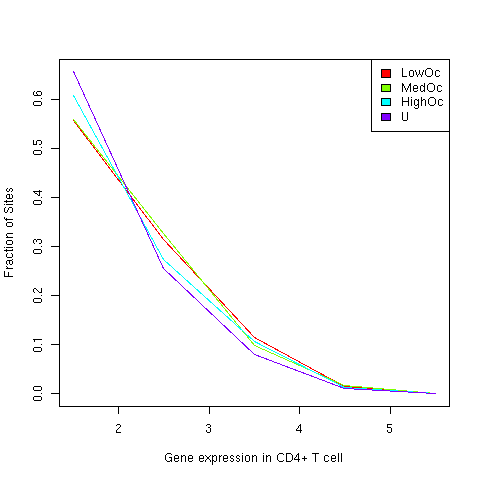  (a) | 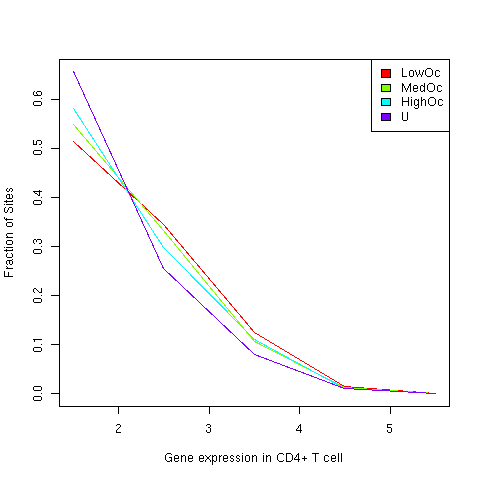  (b) |
| --- | --- |

***Figure S4.*** *Expression distribution for the gene closest to a CTCF site in the three classes of CTCF sites, as well as the unoccupied sites used as a negative control. (a) Using only the sites shared among all 4 cell types, (b) using all sites in CD4+ T cell.*

***Figure S5.*** *Distributions of mouse CTCF sites into the three classes. The total number of sites in each analysis is shown in parentheses. Overall, there are 19.5 % of LowOc sites. However within the 10 kb upstream of genes down-regulated (DN) in CTCF knocked-down oocytes, 24 % of the sites are from the LowOc class, whereas within the 10 kb upstream of genes up-regulated (UP) in CTCF knocked-down oocytes, only 16 % of the sites are from the LowOc class. Enrichment of LowOc near DN genes is statistically significant.*

***Table S1.*** *For each of the histone marks, the table shows the Wilcoxon one-sided rank-sum test p-values for tag density enrichment in ±500 bp flanking one CTCF site class relative to another, as well as relative to the control U sites, in pair-wise fashion. Column headings represent the CTCF sites classes compared, say Mi~Mj. Only the significant* p-values are shown. Bold text indicates the cases where the tag density for Mi was significantly greater than that for Mj, and italicized text indicates the opposite relation. This analysis is the same as shown in Table 1, but here we use all CD4+ T cells CTCF sites, and not just the ones shared between 4 cell types.

|  | **LowOc~MedOc** | **MedOc~HighOc** | **LowOc~HighOc** | **LowOc~U** | **MedOc~U** | **HighOc~U** |
| --- | --- | --- | --- | --- | --- | --- |
| **H3K4me1** | **0** |  | **0** | **0** | **0** | **0** |
| **H3K4me2** | **0** | **0.02** | **0** | **0** | **0** | **0** |
| **H3K4me3** | **0** | **0.02** | **0** | **0** | **0** | **0** |
| **H3K27me1** | **0** | **1.00E-04** | **0** | **0** | **0** | **0** |
| **H3K27me2** | *1.21E-12* | *5.69E-04* | *8.37E-21* | *3.82E-128* | *1.37E-88* | *2.94E-52* |
| **H3K27me3** | *2.21E-15* | *5.35E-06* | *1.03E-28* | *2.94E-56* | *1.50E-22* | *4.66E-07* |
| **H3K36me1** | **1.65E-08** | **0.02055172** | **8.52E-12** | **0** | **0** | **0** |
| **H3K36me3** |  | **8.06E-03** | **0.02** | *3.27E-08* | *5.29E-09* | *8.32E-13* |
| **H3K79me3** | **0** | **1.68E-06** | **0** | **0** | **0** | **0** |
| **H3K9me1** | **0** | **0.01** | **0** | **0** | **0** | **0** |
| **H4K20me1** | **0** |  | **2.22E-16** | **0** | **0** | **0** |
| **H2BK5me1** | **0** |  | **0** | **0** | **0** | **0** |
| **H2AK9ac** | **0** | **9.31E-05** | **0** | **0** | **0** | **0** |
| **H4K12ac** | **0** | **4.66E-07** | **0** | **0** | **0** | **0** |
| **H4K16ac** | **0** | **2.59E-08** | **0** | **0** | **0** | **0** |
| **H2AZ** | **0** |  | **0** | **0** | **0** | **0** |

***Table S2.*** *For each histone mark and for each CTCF site class, the table shows the fraction of sites for which the tags in the ±5 kbp flanking region are unequally distributed between the upstream and the downstream. We estimated the significance of biased distribution using Fisher exact test. While at random, we expect ~5 % of sites to exhibit significantly biased distribution at p-value ≤ 0.05, for most activation sites we see a multi-fold enrichment in this fraction. Moreover, in general, LowOc sites exhibit a larger enrichment. Cases with greater than* 2-fold enrichment are in bold text.

|  | **LowOc** | **MedOc** | **HighOc** |
| --- | --- | --- | --- |
| **H3K4me1** | **0.412** | **0.377** | **0.365** |
| **H3K4me2** | **0.301** | **0.259** | **0.243** |
| **H3K4me3** | **0.399** | **0.34** | **0.321** |
| **H3K27me1** | **0.163** | **0.162** | **0.142** |
| **H3K27me2** | 0.037 | 0.039 | 0.036 |
| **H3K27me3** | 0.083 | 0.091 | 0.089 |
| **H3K36me1** | 0.041 | 0.046 | 0.042 |
| **H3K36me3** | **0.173** | **0.149** | **0.132** |
| **H3K79me3** | **0.171** | **0.124** | **0.106** |
| **H3K9me1** | **0.343** | **0.293** | **0.268** |
| **H4K20me1** | **0.342** | **0.288** | **0.279** |
| **H2BK5me1** | **0.26** | **0.228** | **0.215** |
| **H2AK9ac** | 0.044 | 0.038 | 0.026 |
| **H4K12ac** | 0.072 | 0.056 | 0.054 |
| **H4K16ac** | **0.121** | **0.1** | 0.087 |
| **H2AZ** | **0.25** | **0.226** | **0.217** |

***Table S3.*** For each histone mark and for each CTCF site class, we performed a paired-Wilcoxon test for the alternative hypotheses indicated in the first row. For instance, “LowOc (up<down)” tests whether the tag counts in the upstream of a LowOc site is less than that in the downstream of the same site. Significant p-values are highlighted in bold text.

|  | **LowOc**  **(up<down)** | **LowOc**  **(up>down)** | **MedOc**  **(up<down)** | **MedOc**  **(up>down)** | **HighOc**  **(up<down)** | **HighOc**  **(up>down)** |
| --- | --- | --- | --- | --- | --- | --- |
| **H3K4me1** | **3.94E-06** | 1.000 | **4.50E-16** | 1.000 | **2.81E-17** | 1.000 |
| **H3K4me2** | **1.03E-07** | 1.000 | **7.22E-20** | 1.000 | **6.67E-24** | 1.000 |
| **H3K4me3** | **8.49E-06** | 1.000 | **1.27E-14** | 1.000 | **7.16E-20** | 1.000 |
| **H3K27me1** | **8.23E-05** | 1.000 | **1.02E-09** | 1.000 | **2.90E-09** | 1.000 |
| **H3K27me2** | **2.63E-02** | 0.974 | **2.91E-02** | 0.971 | 0.189 | 0.811 |
| **H3K27me3** | 0.935 | 0.065 | 0.997 | **0.003** | 0.809 | 0.191 |
| **H3K36me1** | 0.111 | 0.889 | **1.99E-05** | 1.000 | **1.66E-04** | 1.000 |
| **H3K36me3** | 0.120 | 0.880 | 0.202 | 0.798 | **1.95E-02** | 0.980 |
| **H3K79me3** | **5.34E-02** | 0.947 | **1.60E-02** | 0.984 | **3.65E-02** | 0.963 |
| **H3K9me1** | **2.29E-06** | 1.000 | **2.05E-22** | 1.000 | **1.55E-23** | 1.000 |
| **H4K20me1** | **1.79E-02** | 0.982 | 0.496 | 0.504 | **4.31E-02** | 0.957 |
| **H2BK5me1** | **6.56E-02** | 0.934 | **2.44E-02** | 0.976 | **1.42E-03** | 0.999 |
| **H2AK9ac** | 0.129 | 0.871 | **1.30E-03** | 0.999 | **2.41E-07** | 1.000 |
| **H4K12ac** | 0.460 | 0.540 | **2.28E-06** | 1.000 | **3.27E-08** | 1.000 |
| **H4K16ac** | **1.19E-03** | 0.999 | **2.02E-04** | 1.000 | **1.18E-04** | 1.000 |
| **H2AZ** | **2.51E-05** | 1.000 | **9.26E-23** | 1.000 | **7.11E-27** | 1.000 |

***Table S4.*** *For each of the histone marks, the table shows the Wilcoxon one-sided rank-sum test p-values for the difference in tag-density-differential in one CTCF site class relative to another, in pair-wise fashion. Column headings show the alternative hypothesis tested. Significant p-values are shown in bold text and marginally significant (0.05 < p-value ≤ 0.1) ones are shown in italicized text. In none of the cases, the opposite hypotheses (respectively, LowOc<MedOc, LowOc<HighOc and MedOc<HighOc ) wa*s significant.

|  | **LowOc>MedOc** | **LowOc>HighOc** | **MedOc>HighOc** |
| --- | --- | --- | --- |
| **H3K4me1** | 0.315 | 0.261 | 0.382 |
| **H3K4me2** | 0.561 | 0.382 | 0.346 |
| **H3K4me3** | 0.382 | 0.102 | 0.150 |
| **H3K27me1** | 0.863 | 0.571 | 0.218 |
| **H3K27me2** | 0.229 | 0.240 | 0.491 |
| **H3K27me3** | *0.086* | **0.044** | 0.420 |
| **H3K36me1** | 0.596 | 0.112 | 0.147 |
| **H3K36me3** | **0.014** | **0.005** | 0.279 |
| **H3K79me3** | 0.532 | 0.606 | 0.478 |
| **H3K9me1** | 0.238 | 0.104 | 0.123 |
| **H4K20me1** | **0.023** | **9.16E-05** | *0.051* |
| **H2BK5me1** | **0.028** | **0.020** | 0.188 |
| **H2AK9ac** | **0.047** | 0.105 | 0.684 |
| **H4K12ac** | 0.108 | 0.234 | 0.652 |
| **H4K16ac** | *0.062* | **0.003** | **0.034** |
| **H2AZ** | 0.700 | 0.545 | 0.512 |

***Table S5.*** Motifs enriched in the 200 bp flanking CTCF bound sites relative to control unoccupied sites. Only motifs with enrichment FDR <= 10 % are shown.

| **Transcription Factor** | **TRANSFAC PWM Id** | **Fold Enrichment** | **Fisher p-**  **value** | **FDR** |
| --- | --- | --- | --- | --- |
| E2F | M00050 | 2.80 | 0 | 0 |
| ZF5 | M00716 | 2.35 | 0 | 0 |
| Nrf-1 | M00652 | 2.18 | 0 | 0 |
| CBF_(core_binding_factor) | M01079 | 1.86 | 0 | 0 |
| HES1 | M01009 | 1.75 | 0 | 0 |
| Whn | M00332 | 1.73 | 0 | 0 |
| DEAF1 | M01002 | 1.61 | 0 | 0 |
| STAT3 | M00497 | 1.59 | 0 | 0 |
| DEAF1 | M01001 | 1.59 | 0 | 0 |
| CBF_(core_binding_factor) | M01080 | 1.56 | 0 | 0 |
| neural-restr.-silencer-element | M00325 | 1.50 | 0 | 0 |
| AhR:Arnt | M00237 | 1.49 | 0 | 0 |
| c-Ets-1_p54 | M01078 | 1.45 | 0 | 0 |
| MTF-1 | M00650 | 1.45 | 0 | 0 |
| LXR | M00647 | 1.43 | 0 | 0 |
| LRF | M01100 | 1.43 | 0 | 0 |
| PTF1-beta | M00657 | 1.38 | 0 | 0 |
| HIC1 | M01073 | 1.35 | 0 | 0 |
| R | M00273 | 1.34 | 0 | 0 |
| HIC1 | M01072 | 1.33 | 0 | 0 |
| Egr-3 | M00245 | 1.32 | 0 | 0 |
| AP-2 | M00915 | 1.18 | 1.00E-06 | 1.02E-05 |
| Muscle_initiator_sequence-20 | M00324 | 1.22 | 3.00E-06 | 2.93E-05 |
| Pax-5 | M00143 | 1.22 | 5.00E-06 | 4.69E-05 |
| MOVO-B | M01104 | 1.45 | 8.00E-06 | 7.20E-05 |
| Ik-2 | M00087 | 1.33 | 9.00E-06 | 7.79E-05 |
| E2 | M00928 | 1.35 | 2.00E-05 | 0.000167 |
| Msx-1 | M00394 | 1.46 | 9.00E-05 | 0.000723 |
| CHOP:C/EBPalpha | M00249 | 1.32 | 0.00012 | 0.000931 |
| Ncx | M00484 | 1.31 | 0.000171 | 0.001283 |
| RFX1 | M00281 | 1.27 | 0.00027 | 0.00196 |
| Hmx3 | M00433 | 1.25 | 0.000315 | 0.002215 |
| Egr-2 | M00246 | 1.20 | 0.000439 | 0.002993 |
| SZF1-1 | M01109 | 1.23 | 0.000604 | 0.003997 |
| C/EBPdelta | M00621 | 1.41 | 0.000771 | 0.004944 |
| CACCC-binding_factor | M00721 | 1.16 | 0.000791 | 0.004944 |
| Roaz | M00467 | 1.21 | 0.000881 | 0.005288 |
| Churchill | M00986 | 1.18 | 0.000893 | 0.005288 |
| TFIIA | M00707 | 1.27 | 0.001014 | 0.00585 |
| p300 | M00033 | 1.18 | 0.001405 | 0.007903 |
| EGR | M00807 | 1.13 | 0.00268 | 0.014707 |
| STAT1 | M00496 | 1.27 | 0.002854 | 0.015289 |
| Pax-5 | M00144 | 1.22 | 0.003509 | 0.018361 |
| Ik-1 | M00086 | 1.19 | 0.003658 | 0.018706 |
| WT1 | M01118 | 1.17 | 0.004109 | 0.020545 |
| Gfi-1 | M00250 | 1.28 | 0.005514 | 0.026971 |
| HMGIY | M01010 | 1.36 | 0.006159 | 0.029485 |
| ZID | M00085 | 1.15 | 0.006522 | 0.030572 |
| v-Myb | M00003 | 1.23 | 0.006668 | 0.030618 |
| AP-2alphaA | M01047 | 1.19 | 0.007563 | 0.034034 |
| RFX1_(EF-C) | M00626 | 1.25 | 0.007811 | 0.03446 |
| Sp1 | M00931 | 1.12 | 0.008057 | 0.034862 |
| Pax-2 | M00098 | 1.19 | 0.008854 | 0.037588 |
| XPF-1 | M00684 | 1.15 | 0.011683 | 0.048679 |
| STAT4 | M00498 | 1.28 | 0.012839 | 0.052523 |
| AP-2alphaA | M01045 | 1.09 | 0.01698 | 0.068223 |
| HOXA3 | M00395 | 1.20 | 0.021257 | 0.083909 |
| STAT5A | M00499 | 1.19 | 0.021901 | 0.084961 |

***Table S6****.*Functional enrichment near each of the three CTCF site classes, relative to all genes near any CTCF site. We used a FDR threshold of 20 %.

| **Term** | **Fold Enrichment** | **FDR** | ***P-value*** |
| --- | --- | --- | --- |
| **LowOc mode** | | | |
| icosanoid metabolic process | 2.85 | 2.80E-03 | 1.50E-04 |
| carboxylic acid metabolic process | 1.41 | 2.90E-02 | 1.59E-03 |
| organic acid metabolic process | 1.40 | 3.47E-02 | 1.91E-03 |
| icosanoid biosynthetic process | 2.87 | 5.32E-02 | 2.96E-03 |
| membrane lipid metabolic process | 1.61 | 0.102 | 5.85E-03 |
| macromolecule metabolic process | 1.07 | 0.123 | 7.09E-03 |
| organelle organization and biogenesis | 1.22 | 0.139 | 8.10E-03 |
| alkene metabolic process | 3.07 | 0.146 | 8.52E-03 |
| leukotriene metabolic process | 3.07 | 0.146 | 8.52E-03 |
| membrane lipid biosynthetic process | 1.95 | 0.148 | 8.64E-03 |
| regulation of growth | 1.52 | 0.149 | 8.69E-03 |
| amino acid metabolic process | 1.48 | 0.157 | 9.22E-03 |
| lipid biosynthetic process | 1.47 | 0.188 | 1.12E-02 |
| **MedOc mode** | | | |
| pos reg of multicellular organismal process | 1.43 | 9.22E-02 | 5.23E-03 |
| **HighOc mode** | | | |
| neurite development | 1.53 | 1.55E-02 | 8.45E-04 |
| cell projection organization and biogenesis | 1.41 | 4.09E-02 | 2.27E-03 |
| cell projection morphogenesis | 1.41 | 4.09E-02 | 2.27E-03 |
| cell part morphogenesis | 1.41 | 4.09E-02 | 2.27E-03 |
| neuron morphogenesis during differentiation | 1.48 | 5.25E-02 | 2.92E-03 |
| neurite morphogenesis | 1.48 | 5.25E-02 | 2.92E-03 |
| cellular morphogenesis during differentiation | 1.47 | 5.36E-02 | 2.99E-03 |
| neuron development | 1.43 | 5.70E-02 | 3.18E-03 |
| neuron differentiation | 1.34 | 0.10 | 5.74E-03 |
| axonogenesis | 1.45 | 0.11 | 6.36E-03 |
| actin filament-based process | 1.38 | 0.11 | 6.37E-03 |

***Table S7.*** *For each CTCF class and for each enriched functional category for that CTCF class, we tested, using the Wilcoxon one-sided test, whether the specific genes (FG) have unusually higher or lower expression than all other genes (BG), in CD4+ T cell. Certain functional classes in S6 have been eliminated as they corresponded to identical sets of genes in another functional class. Column 2 shows the number of genes with expression data available. Columns 3 and 4 show the median expression for FG and BG. The last two columns show the p-values for the alternative hypothesis shown in the header row. Significant p-values are shown in bold.*

| **GO category** | **#Genes** | **FG med expr** | **BG med expr** | **FG > BG** | **FG < BG** |
| --- | --- | --- | --- | --- | --- |
| **LowOc** | | | | | |
| icosanoid metabolic process | 9 | 2.62 | 2.36 | 0.231 | 0.769 |
| carboxylic acid metabolic process | 37 | 2.30 | 2.36 | 0.739 | 0.261 |
| icosanoid biosynthetic process | 6 | 2.54 | 2.36 | 0.318 | 0.682 |
| membrane lipid metabolic process | 18 | 2.17 | 2.36 | 0.723 | 0.277 |
| macromolecule metabolic process | 252 | 2.55 | 2.35 | **0.001** | 0.999 |
| organelle organization and biogenesis | 51 | 2.69 | 2.36 | **0.00024** | 1.000 |
| alkene metabolic process | 5 | 2.42 | 2.36 | 0.575 | 0.425 |
| membrane lipid biosynthetic process | 8 | 2.55 | 2.36 | 0.397 | 0.603 |
| regulation of growth | 20 | 2.32 | 2.36 | 0.771 | 0.229 |
| amino acid metabolic process | 17 | 2.26 | 2.36 | 0.763 | 0.237 |
| lipid biosynthetic process | 20 | 2.44 | 2.36 | 0.517 | 0.483 |
| **MedOc** | | | | | |
| positive reg of multicellular org process | 16 | 2.00 | 2.36 | 0.994 | 0.006 |
| **HighOc** | | | | | |
| neurite development | 17 | 2.20 | 2.36 | 0.920 | 0.080 |
| Cell projection organization and biogenesis | 22 | 2.22 | 2.36 | 0.856 | 0.144 |
| Cell morphogenesis during differentiation | 15 | 2.20 | 2.36 | 0.866 | 0.134 |
| neuron development | 20 | 2.22 | 2.36 | 0.960 | **0.040** |
| neuron differentiation | 23 | 2.23 | 2.36 | 0.912 | 0.088 |
| axonogenesis | 14 | 2.19 | 2.36 | 0.909 | 0.091 |
| actin filament-based process | 15 | 2.29 | 2.36 | 0.194 | 0.806 |

*Table S8. The table shows the enriched k-mers in each of the three CTCF classes relative to the other two classes. The analysis was done separately for the 2 conserved cores in the CTCF motif – a 5-mer core spanning positions 4-8 and a 9-mer core spanning positions 10-18. Only the k-mers that were enriched with a false discovery rate of 1% or less are shown.*

| **Pattern** | **Enrichment P-value** | **Q-value** |
| --- | --- | --- |
| **The first 5-mer core** | | |
|
| **LowOc** | | |
| TGCAC | 1.95E-007 | 4.93E-006 |
| TTCAC | 9.17E-007 | 2.15E-005 |
| TCCCC | 1.73E-006 | 3.35E-005 |
| CACAC | 4.31E-006 | 7.07E-005 |
| TGCAG | 4.31E-006 | 7.07E-005 |
| TCCAC | 4.53E-006 | 7.08E-005 |
| CTCAC | 2.02E-005 | 0.000245897 |
| TACAC | 2.02E-005 | 0.000245897 |
| TACAG | 2.02E-005 | 0.000245897 |
| TTCAG | 2.02E-005 | 0.000245897 |
| AACGC | 3.14E-005 | 0.000368751 |
| TCCAT | 9.48E-005 | 0.001004737 |
| CCCAC | 0.00012632 | 0.001296672 |
| AACAC | 0.000294772 | 0.002799583 |
| CCTGC | 0.000444612 | 0.003947176 |
| **MedOc** | | |
| TCCAC | 2.62E-013 | 1.72E-011 |
| GTCAG | 4.08E-012 | 2.23E-010 |
| TCCAG | 1.03E-010 | 4.83E-009 |
| CCCAC | 4.13E-010 | 1.70E-008 |
| CCCAG | 1.41E-008 | 4.62E-007 |
| GACAC | 1.32E-007 | 3.94E-006 |
| GACAG | 1.92E-007 | 4.93E-006 |
| GTCAC | 1.05E-006 | 2.30E-005 |
| GGCAC | 6.54E-005 | 0.000715961 |
| CCCTC | 0.000274957 | 0.002736888 |
| ATCAC | 0.000298301 | 0.002799583 |
| AACAC | 0.001129467 | 0.009763304 |
| **HighOc** | | |
| GCCAG | 4.11E-068 | 1.35E-065 |
| GCCAC | 2.07E-048 | 3.40E-046 |
| ACCAC | 6.50E-022 | 7.12E-020 |
| ACCAG | 9.53E-022 | 7.83E-020 |
| GCCGG | 1.27E-008 | 4.62E-007 |
| ACCTG | 1.65E-006 | 3.35E-005 |
| GCCTC | 2.92E-006 | 5.32E-005 |
| ACCTC | 5.71E-006 | 8.52E-005 |
| GCCTG | 6.75E-006 | 9.65E-005 |
| ACCGG | 4.00E-005 | 0.000452519 |
| GCCGC | 0.000426985 | 0.003895985 |
| **The second 9-mer core** | | |
|
| **LowOc** | | |
| AGGGGGCCT | 1.95E-007 | 1.09E-005 |
| AGGGGGAGA | 5.37E-006 | 0.000212738 |
| AGATGGCGT | 0.00019571 | 0.005424125 |
| AGGGGGCCA | 0.000444612 | 0.00926503 |
| GGGAGGCGC | 0.000444612 | 0.00926503 |
| **MedOc** | | |
| AGAGGACAG | 0.000129947 | 0.003791037 |
| GGGTGGCAG | 0.000265748 | 0.007014522 |
| AGGTGTCAG | 0.000493442 | 0.009768443 |
| **HighOc** | | |
| AGGGGGCAG | 3.00E-018 | 1.66E-015 |
| AGGTGGCAG | 8.82E-018 | 2.45E-015 |
| AGAGGGCAG | 2.89E-017 | 5.34E-015 |
| AGGGGGCGC | 9.82E-013 | 1.36E-010 |
| AGAGGGCGC | 3.06E-011 | 3.40E-009 |
| AGGTGGCAC | 2.51E-009 | 2.32E-007 |
| AGATGGCAG | 4.99E-009 | 3.95E-007 |
| AGGTGGCGC | 4.67E-008 | 3.24E-006 |
| AGGGGGCAC | 1.97E-007 | 1.09E-005 |
| AGATGGCAC | 2.22E-007 | 1.12E-005 |
| AGAGGGAGC | 1.49E-006 | 6.90E-005 |
| AGAGGGCAC | 4.71E-006 | 0.000200971 |
| AGGGGGTGC | 1.94E-005 | 0.000715507 |
| AGGTGGCGG | 5.23E-005 | 0.00170626 |
| AGGTGGTGC | 5.04E-005 | 0.00170626 |
| AGGGGGCGG | 9.32E-005 | 0.002869817 |
| AGATGGCGG | 0.000322546 | 0.008126734 |
| AGGTGGCTC | 0.000353301 | 0.008514583 |
| AGGGGGAGC | 0.000396044 | 0.00914701 |
| AGAGGGCGG | 0.000451298 | 0.00926503 |

Table S9. The table summarizes the main observed differences between the LowOc and HighOc CTCF binding site classes with respect to the various types of features studied.

|  | **LowOc** | **MedOc** | **HighOc** |
| --- | --- | --- | --- |
| **Binding site sequence** | Lower PWM score, higher number of specific k-mers | Intermediate PWM score, intermediate number of specific k-mers | Higher PWM score, lower number of specific k-mers |
| **CTCF binding** | Lower ChIP-seq tag count, higher cell-type specificity | Intermediate ChIP-seq tag count, intermediate cell-type specificity | Higher ChIP-seq tag count, lower cell-type specificity |
| **Associated genomic features** | Clustering, lower GC content, farther to interspersed repeats and low complexity DNA, association with TSS, association with POU6F1 binding motif, association with genes involved in several metabolic processes | Lower GC content, farther to interspersed repeats and low complexity DNA, association with genes involved in the positive regulation of multicellular organismal processes | Higher GC content, closer to interspersed repeats and low complexity DNA, association with several transcription factors binding motifs including YY1, association with genes involved in cell and neuronal morphogenesis and in neuronal differentiation |
| **Evolution** | Lower conservation of binding site, higher conservation of flanking sequences, low rate of mutation into MedOc and HighOc sites between human and mouse | Intermediate conservation of binding site, intermediate conservation of flanking sequences, tend to remain MedOc sites between human and mouse, low rate of mutation into LowOc site between human and mouse | Higher conservation of binding site, lower conservation of flanking sequences, tend to remain HighOc sites between human and mouse, low rate of mutation into LowOc site between human and mouse |
| **Gene expression levels** | Association with higher gene expression, association with downregulated genes in CTCF knock-down oocytes |  | Associated with lower gene expression |
| **Gene expression differences** | Higher differential of expression from flanking divergent promoters, higher difference of expression within CTCF flanked blocks | Lower differential of expression from flanking divergent promoters, higher difference of expression within CTCF flanked blocks | Lower differential of expression from flanking divergent promoters, lower difference of expression within CTCF flanked blocks |
| **Histone marks density** | Higher for most euchromatic marks, lower for heterochromatic marks | Lower for a majority of euchromatic marks, intermediate for heterochromatic marks | Lower for euchromatic marks, higher for heterochromatic marks |
| **Unequal distribution of histone marks between flanks** | Higher for several euchromatic marks, higher for H3K27me3 (heterochromatic mark) | Lower or intermediate for several euchromatic marks, lower for H3K27me3 (heterochromatic mark) | Lower for several heterochromatic marks, lower for H3K27me3 (heterochromatic mark) |
| **Side of higher histone mark enrichment between flanks** | Downstream for a majority of euchromatic marks, downstream for H3K27me2 (heterochromatic mark) | Downstream for most euchromatic marks, downstream for H3K27me2 but upstream for H3K27me3 (heterochromatic marks) | Downstream for euchromatic marks |
