# Supplementary material for: CTCF binding site classes exhibit distinct evolutionary, genomic, epigenomic and transcriptomic features
Source: Genome Biol. 2009 Nov 18;10(11):R131. doi: 10.1186/gb-2009-10-11-r131 (PMC3091324; doi:10.1186/gb-2009-10-11-r131)
Supplement: Additional data file 3 — Experimentally determined CTCF sites in human and mouse and their occupancy classes. [file gb-2009-10-11-r131-S3.DOC]

**PWM score and occupancy class of CTCF sites reported in the literature**

For each mentioned site, the highest PWM score was determined from the published binding site sequence or from the smallest CTCF-binding fragment reported in the literature. The corresponding CTCF class is indicated, as well as the position of the CTCF site. The methods used for the identification of CTCF sites are indicated, with EMSA, Electrophoretic Mobility Shift Assay, and ChIP, Chromatin Immunoprecipitation. The CTCF sites activity is indicated even when it has been reported only for sequences larger than the CTCF site, sometimes also encompassing other CTCF sites. More precise information can be found in the indicated references. Depending on the loci, the functional link between CTCF binding and the biological processes mentioned in the last column are either demonstrated or hypothesized.

1. Human sites

| **Locus** | **Site name** | **Coordinate (UCSC Mar 2006)** | **PWM score** | **Class** | **Method of identification** | **Associated activity** | **Associated biological process** |
| --- | --- | --- | --- | --- | --- | --- | --- |
| APOB | APOB | chr2:21174444-21174463 | 0.80651 | M1 | EMSA, ChIP (Biochemistry. 2001 Jun 12;40(23):6731-42.) | Enhancer-blocking (Biochemistry. 2001 Jun 12;40(23):6731-42.) |  |
| IRAK2 | IRAK2 prom. | chr3:10181499-10181518 | 0.77714 | NA | EMSA, ChIP (J Mol Biol. 2005 Feb 18;346(2):411-22.) | Transcriptional activation (J Mol Biol. 2005 Feb 18;346(2):411-22.) |  |
| SCA7 | SCA7-CTCF-II | chr3:63873352-63873371 | 0.83376 | M1 | EMSA (Nat Genet. 2001 Aug;28(4):335-43.; PLoS Genet. 2008 Nov;4(11):e1000257.), DNAse footprint, ChIP (PLoS Genet. 2008 Nov;4(11):e1000257.) | Triplet-repeat stability (PLoS Genet. 2008 Nov;4(11):e1000257.) | CTG/CAG repeats expansion |
|  | SCA7-CTCF-I | chr3:63873723-63873742 | 0.86846 | M2 | EMSA (Nat Genet. 2001 Aug;28(4):335-43.; PLoS Genet. 2008 Nov;4(11):e1000257.), DNAse footprint, ChIP (PLoS Genet. 2008 Nov;4(11):e1000257.) | Triplet-repeat stability (PLoS Genet. 2008 Nov;4(11):e1000257.) | CTG/CAG repeats expansion |
| IL3 | IL-3 +2.9kb DHS | chr5:131427206-131427225 | 0.89246 | M2 | EMSA, DNAse footprint, ChIP (Mol Cell Biol. 2009 Apr;29(7):1682-93.) | Enhancer-blocking (Mol Cell Biol. 2009 Apr;29(7):1682-93.) | T-cell development |
|  | IL-3 +4.2kb DHS | chr5:131428454-131428473 | 0.95215 | M3 | EMSA, DNAse footprint, ChIP (Mol Cell Biol. 2009 Apr;29(7):1682-93.) | Enhancer-blocking (Mol Cell Biol. 2009 Apr;29(7):1682-93.) | T-cell development |
|  | IL-3 +4.9kb DHS | chr5:131429134-131429153 | 0.84549 | M1 | EMSA, DNAse footprint, ChIP (Mol Cell Biol. 2009 Apr;29(7):1682-93.) | Enhancer-blocking (Mol Cell Biol. 2009 Apr;29(7):1682-93.) | T-cell development |
| hTERT | hTERT proximal exonic region F3 | chr5:1347608-1347627 | 0.84327 | M1 | EMSA, ChIP (Nucleic Acids Res. 2005 Dec 2;33(21):6850-60.) | Transcriptional repression (Nucleic Acids Res. 2005 Dec 2;33(21):6850-60.) |  |
|  | hTERT proximal exonic region F1 | chr5:1347997-1348016 | 0.89862 | M2 | EMSA, ChIP (Nucleic Acids Res. 2005 Dec 2;33(21):6850-60.) | Transcriptional repression (Nucleic Acids Res. 2005 Dec 2;33(21):6850-60.) |  |
| MHC-II | XL9-d | chr6:32698455-32698474 | 0.76138 | NA | EMSA, DNAse footprint, ChIP (J Biol Chem. 2006 Jul 7;281(27):18435-43.) | Enhancer-blocking (J Biol Chem. 2006 Jul 7;281(27):18435-43.), gene activation, long-range interactions (J Exp Med. 2008 Apr 14;205(4):785-98.) | B-cell development |
| PIM1 | PIM1 prom. | chr6:37245581-37245600 | 0.89381 | M2 | EMSA (Cancer Res. 2002 Jan 1;62(1):48-52.) | ND |  |
| MEST/MESTIT1 | MEST prom. | chr7:129913632-129913651 | 0.83077 | M1 | ChIP-seq (Cell. 2007 May 18;129(4):823-37.) | ND | Imprinting |
| PEG10/SGCE | PEG10 prom. | chr7:94123457-94123476 | 0.8806 | M2 | ChIP-seq (Cell. 2007 May 18;129(4):823-37.) | ND | Imprinting |
| MYC | MYC I | chr8:128811772-128811791 | 0.82096 | M1 | Sequence only (Mol Cell Biol. 2003 Dec;23(24):9338-48.) | ND |  |
|  | MYC II, 5' boundary, site N | chr8:128815533-128815552 | 0.9591 | M3 | ChIP (Mol Cell Biol. 2003 Dec;23(24):9338-48.) | Not required for proper MYC expression (PLoS One. 2009 Jul 1;4(7):e6109.) |  |
|  | MYC III | chr8:128817384-128817403 | 0.75318 | NA | Sequence only (Mol Cell Biol. 2003 Dec;23(24):9338-48.) | ND |  |
|  | MYC IV, site B (P1) | chr8:128817520-128817539 | 0.84711 | M1 | EMSA assay (Mol Cell Biol. 1996 Jun;16(6):2802-13.) | ND |  |
|  | MYC V, site A (P2) | chr8:128817679-128817698 | 0.82258 | M1 | EMSA assay (Mol Cell Biol. 1996 Jun;16(6):2802-13.), ChIP (Mol Cell Biol. 2003 Dec;23(24):9338-48.) | Transcriptional repression (Mol Cell Biol. 1996 Jun;16(6):2802-13.), enhancer-blocking (Mol Cell Biol. 2003 Dec;23(24):9338-48.), transcriptional activation (PLoS One. 2009 Jul 1;4(7):e6109.) |  |
| BAG1 | BAG1 prom. site 3 | chr9:33254955-33254974 | 0.74894 | NA | EMSA, ChIP (Cancer Res. 2008 Apr 15;68(8):2726-35.) | Transcriptional repression (Cancer Res. 2008 Apr 15;68(8):2726-35.) |  |
|  | BAG1 prom. site 2 | chr9:33255052-33255071 | 0.63366 | NA | EMSA, ChIP (Cancer Res. 2008 Apr 15;68(8):2726-35.) | Transcriptional repression (Cancer Res. 2008 Apr 15;68(8):2726-35.) |  |
|  | BAG1 prom. site 1 | chr9:33255113-33255132 | 0.69407 | NA | EMSA, ChIP (Cancer Res. 2008 Apr 15;68(8):2726-35.) | Transcriptional repression (Cancer Res. 2008 Apr 15;68(8):2726-35.) |  |
| H19/MRPPL23 | hPCT14 | chr11:1944499-1944518 | 0.83273 | M1 | EMSA (Hum Mol Genet. 2002 Jul 1;11(14):1627-36.) | ND |  |
|  | hPCT12 | chr11:1947038-1947057 | 0.88576 | M2 | EMSA (Hum Mol Genet. 2002 Jul 1;11(14):1627-36.) | ND |  |
| H19 | h7 | chr11:1976861-1976880 | 0.76505 | NA | EMSA (Nature. 2000 May 25;405(6785):486-9.) | Enhancer-blocking (Hum Mol Genet. 2003 Aug 1;12(15):1927-39.) | Imprinting |
|  | h2 | chr11:1977788-1977807 | 0.83588 | M1 | EMSA (Hum Mol Genet. 2003 Aug 1;12(15):1927-39.) | Enhancer-blocking (Hum Mol Genet. 2003 Aug 1;12(15):1927-39.) | Imprinting |
|  | h5 | chr11:1978194-1978213 | 0.77006 | NA | EMSA (Hum Mol Genet. 2003 Aug 1;12(15):1927-39.) | Enhancer-blocking (Hum Mol Genet. 2003 Aug 1;12(15):1927-39.) | Imprinting |
|  | h3 | chr11:1978600-1978619 | 0.83588 | M1 | Sequence only (Nature. 2000 May 25;405(6785):482-5.) | Enhancer-blocking (Hum Mol Genet. 2003 Aug 1;12(15):1927-39.) | Imprinting |
|  | h4 | chr11:1980033-1980052 | 0.83588 | M1 | Sequence only (Nature. 2000 May 25;405(6785):482-5.) | Enhancer-blocking (Hum Mol Genet. 2003 Aug 1;12(15):1927-39.) | Imprinting |
|  | h6 | chr11:1980432-1980451 | 0.76093 | NA | Sequence only (Nature. 2000 May 25;405(6785):482-5.) | Enhancer-blocking (Hum Mol Genet. 2003 Aug 1;12(15):1927-39.) | Imprinting |
|  | h1 | chr11:1980839-1980858 | 0.83588 | M1 | Sequence only (Nature. 2000 May 25;405(6785):482-5.) | Enhancer-blocking (Hum Mol Genet. 2003 Aug 1;12(15):1927-39.) | Imprinting |
| IGF2 | IGF2-CBi | chr11:2112114-2112133 | 0.88516 | M2 | EMSA (Hum Mol Genet. 2003 Aug 1;12(15):1927-39.) | Enhancer-blocking (Hum Mol Genet. 2003 Aug 1;12(15):1927-39.) | Imprinting |
|  | IGF2-DBi | chr11:2118384-2118403 | 0.88983 | M2 | EMSA (Hum Mol Genet. 2003 Aug 1;12(15):1927-39.) | Enhancer-blocking (Hum Mol Genet. 2003 Aug 1;12(15):1927-39.) | Imprinting |
| KvDMR | KvDMR-GBi | chr11:2677550-2677569 | 0.78138 | NA | EMSA (Hum Mol Genet. 2003 Aug 1;12(15):1927-39.) | Enhancer-blocking (Hum Mol Genet. 2003 Aug 1;12(15):1927-39.) | Imprinting |
|  | KvDMR-HBi | chr11:2677755-2677774 | 0.8726 | M2 | EMSA (Hum Mol Genet. 2003 Aug 1;12(15):1927-39.) | Enhancer-blocking (Hum Mol Genet. 2003 Aug 1;12(15):1927-39.) | Imprinting |
|  | KvDMR-FBi | chr11:2678190-2678209 | 0.82729 | M1 | EMSA (Hum Mol Genet. 2003 Aug 1;12(15):1927-39.) | Enhancer-blocking (Hum Mol Genet. 2003 Aug 1;12(15):1927-39.) | Imprinting |
| CDKN1C | CDK-LBi | chr11:2862149-2862168 | 0.95902 | M3 | EMSA (Hum Mol Genet. 2003 Aug 1;12(15):1927-39.) | Enhancer-blocking, transcriptional repression (Hum Mol Genet. 2003 Aug 1;12(15):1927-39.) | Imprinting |
|  | CDK-KBi | chr11:2862257-2862276 | 0.82715 | M1 | EMSA (Hum Mol Genet. 2003 Aug 1;12(15):1927-39.) | Enhancer-blocking, transcriptional repression (Hum Mol Genet. 2003 Aug 1;12(15):1927-39.) | Imprinting |
|  | CDK-JBi | chr11:2862487-2862506 | 0.82828 | M1 | EMSA (Hum Mol Genet. 2003 Aug 1;12(15):1927-39.) | Enhancer-blocking, transcriptional repression (Hum Mol Genet. 2003 Aug 1;12(15):1927-39.) | Imprinting |
|  | CDK-IBi | chr11:2863099-2863118 | 0.90633 | M2 | EMSA (Hum Mol Genet. 2003 Aug 1;12(15):1927-39.) | Enhancer-blocking, transcriptional repression (Hum Mol Genet. 2003 Aug 1;12(15):1927-39.) | Imprinting |
| CDKN1C | CDK-HBi | chr11:2864080-2864099 | 0.84412 | M1 | EMSA (Hum Mol Genet. 2003 Aug 1;12(15):1927-39.) | Enhancer-blocking, transcriptional repression (Hum Mol Genet. 2003 Aug 1;12(15):1927-39.) | Imprinting |
| BETA-GLOBIN | 3'HS1 | chr11:5182768-5182787 | 0.82883 | M1 | EMSA (Mol Cell Biol. 2002 Jun;22(11):3820-31.) | Enhancer-blocking (Mol Cell Biol. 2002 Jun;22(11):3820-31.), long-range interactions (Nat Genet. 2003 Oct;35(2):190-4.) |  |
|  | 5'HS5 | chr11:5269239-5269258 | 0.93778 | M3 | EMSA (Mol Cell Biol. 2002 Jun;22(11):3820-31.) | Enhancer-blocking (Mol Cell Biol. 2002 Jun;22(11):3820-31.), long-range interactions (Nat Genet. 2003 Oct;35(2):190-4.) |  |
|  | HS-111 | chr11:5358734-5358753 | 0.95202 | M3 | Sequence only (Mol Cell Biol. 2003 Aug;23(15):5234-44.) | ND |  |
| ATN1 | ATN1-I | chr12:6916076-6916095 | 0.88218 | M2 | EMSA (Nat Genet. 2001 Aug;28(4):335-43.) | ND | CTG/CAG repeats expansion |
|  | ATN1-II | chr12:6916718-6916737 | 0.90011 | M2 | EMSA (Nat Genet. 2001 Aug;28(4):335-43.) | ND | CTG/CAG repeats expansion |
| RB1 | Rb-CTCF | chr13:47775796-47775815 | 0.75257 | NA | EMSA, ChIP (Cancer Res. 2007 Mar 15;67(6):2577-85.) | Transcriptional activation (Cancer Res. 2007 Mar 15;67(6):2577-85.) |  |
| DLK1/GTL2 | MEG3 A | chr14:100355924-100355943 | 0.69299 | NA | EMSA (Chromosome Res. 2005;13(8):809-18.) | ND | Imprinting |
|  | MEG3 B | chr14:100360137-100360156 | 0.71045 | NA | EMSA (Chromosome Res. 2005;13(8):809-18.) | ND | Imprinting |
|  | MEG3 C | chr14:100361304-100361323 | 0.75922 | NA | EMSA, ChIP (Chromosome Res. 2005;13(8):809-18.) | ND | Imprinting |
|  | MEG3 D | chr14:100361832-100361851 | 0.81216 | M1 | EMSA (Chromosome Res. 2005;13(8):809-18.) | ND | Imprinting |
|  | GTL2(a)/MEG3 E | chr14:100364028-100364047 | 0.76625 | NA | EMSA (Chromosome Res. 2005;13(8):809-18.) | ND | Imprinting |
|  | MEG3 F | chr14:100364255-100364274 | 0.76985 | NA | EMSA (Chromosome Res. 2005;13(8):809-18.) | ND | Imprinting |
|  | GTL2(b) | chr14:100364320-100364339 | 0.89541 | M2 | Sequence only (Genome Res. 2000 Nov;10(11):1711-8.) | ND |  |
| TCR | BEAD-1 | chr14:22010524-22010543 | 0.90081 | M2 | EMSA (Cell. 1999 Aug 6;98(3):387-96.) | Enhancer-blocking (Cell. 1999 Aug 6;98(3):387-96.) | T-cell development |
| PLK1 | PLK prom. | chr16:23597378-23597397 | 0.82446 | M1 | EMSA (Cancer Res. 2002 Jan 1;62(1):48-52.) | ND |  |
| BRCA1 | BRCA CTCF#2 | chr17:38531346-38531365 | 0.67994 | NA | EMSA (Int J Cancer. 2004 Sep 20;111(5):669-78), ChIP (Int J Cancer. 2004 Sep 20;111(5):669-78; Breast Cancer Res Treat. 2009 May 23.) | ND |  |
|  | BRCA CTCF#1 | chr17:38531976-38531995 | 0.78279 | NA | EMSA (Int J Cancer. 2004 Sep 20;111(5):669-78), ChIP (Int J Cancer. 2004 Sep 20;111(5):669-78; Breast Cancer Res Treat. 2009 May 23.) | ND |  |
| FXYD5 -COX7A1 | FXYD5-COX7A1 4 | chr19:40475301-40475320 | 0.86018 | M1 | EMSA, ChIP (Anal Biochem. 2006 Jul 1;354(1):85-93.) | ND |  |
|  | FXYD5-COX7A1 3 | chr19:40478201-40478220 | 0.80516 | M1 | EMSA, ChIP (Anal Biochem. 2006 Jul 1;354(1):85-93.) | ND |  |
|  | FXYD5-COX7A1 5 | chr19:40496701-40496720 | 0.83067 | M1 | EMSA, ChIP (Anal Biochem. 2006 Jul 1;354(1):85-93.) | ND |  |
|  | FXYD5-COX7A1 6 | chr19:40511942-40511961 | 0.85943 | M1 | EMSA, ChIP (Anal Biochem. 2006 Jul 1;354(1):85-93.) | ND |  |
|  | FXYD5-COX7A1 9 | chr19:40558760-40558779 | 0.81917 | M1 | EMSA, ChIP (Anal Biochem. 2006 Jul 1;354(1):85-93.) | ND |  |
|  | FXYD5-COX7A1 1 | chr19:40727803-40727822 | 0.77668 | NA | EMSA, ChIP (Anal Biochem. 2006 Jul 1;354(1):85-93.) | ND |  |
|  | FXYD5-COX7A1 8 | chr19:40731544-40731563 | 0.78308 | NA | EMSA, ChIP (Anal Biochem. 2006 Jul 1;354(1):85-93.) | ND |  |
|  | FXYD5-COX7A1 7 | chr19:40732999-40733018 | 0.77225 | NA | EMSA, ChIP (Anal Biochem. 2006 Jul 1;354(1):85-93.) | ND |  |
|  | FXYD5-COX7A1 2 | chr19:40814703-40814722 | 0.79753 | M1 | EMSA, ChIP (Anal Biochem. 2006 Jul 1;354(1):85-93.) | ND |  |
|  | FXYD5-COX7A1 10 | chr19:41287012-41287031 | 0.77333 | NA | EMSA, ChIP (Anal Biochem. 2006 Jul 1;354(1):85-93.) | ND |  |
| DM1 | DM2 site 2 | chr19:50965170-50965189 | 0.80035 | M1 | EMSA, DNAse footprint, ChIP (Nat Genet. 2001 Aug;28(4):335-43.) | Enhancer-blocking (Nat Genet. 2001 Aug;28(4):335-43.) | CTG/CAG repeats expansion |
|  | DM1 site 1 | chr19:50965429-50965448 | 0.96827 | M3 | EMSA, DNAse footprint, ChIP (Nat Genet. 2001 Aug;28(4):335-43.) | Enhancer-blocking (Nat Genet. 2001 Aug;28(4):335-43.) | CTG/CAG repeats expansion |
| BORIS | BORIS prom. C 10 | chr20:55533381-55533400 | 0.76851 | NA | EMSA, ChIP (Biochemistry. 2001 Jun 12;40(23):6731-42.) | Transcriptional repression (Nucleic Acids Res. 2007;35(21):7372-88.) |  |
|  | BORIS prom. C 9 | chr20:55533458-55533477 | 0.76851 | NA | EMSA, ChIP (Biochemistry. 2001 Jun 12;40(23):6731-42.) | Transcriptional repression (Nucleic Acids Res. 2007;35(21):7372-88.) |  |
| BORIS | BORIS prom. C 8 | chr20:55533517-55533536 | 0.84167 | M1 | EMSA, ChIP (Biochemistry. 2001 Jun 12;40(23):6731-42.) | Transcriptional repression (Nucleic Acids Res. 2007;35(21):7372-88.) |  |
|  | BORIS prom. C 6 | chr20:55533813-55533832 | 0.82108 | M1 | EMSA, ChIP (Biochemistry. 2001 Jun 12;40(23):6731-42.) | Transcriptional repression (Nucleic Acids Res. 2007;35(21):7372-88.) |  |
|  | BORIS prom. C 5 | chr20:55533827-55533846 | 0.86415 | M1 | EMSA, ChIP (Biochemistry. 2001 Jun 12;40(23):6731-42.) | Transcriptional repression (Nucleic Acids Res. 2007;35(21):7372-88.) |  |
|  | BORIS prom. C 4 | chr20:55534058-55534077 | 0.86415 | M1 | EMSA, ChIP (Biochemistry. 2001 Jun 12;40(23):6731-42.) | Transcriptional repression (Nucleic Acids Res. 2007;35(21):7372-88.) |  |
|  | BORIS prom. C 3 | chr20:55534207-55534226 | 0.84051 | M1 | EMSA, ChIP (Biochemistry. 2001 Jun 12;40(23):6731-42.) | Transcriptional repression (Nucleic Acids Res. 2007;35(21):7372-88.) |  |
|  | BORIS prom. C 2 | chr20:55534249-55534268 | 0.90141 | M2 | EMSA, ChIP (Biochemistry. 2001 Jun 12;40(23):6731-42.) | Transcriptional repression (Nucleic Acids Res. 2007;35(21):7372-88.) |  |
| GNAS/GNASAS | GNAS prom. | chr20:56861310-56861329 | 0.91611 | M2 | ChIP-seq (Cell. 2007 May 18;129(4):823-37.) | ND | Imprinting |
| APPbeta | APPbeta prom. | chr21:26465031-26465050 | 0.87712 | M2 | EMSA (J Biol Chem. 1997 Dec 26;272(52):33353-9.; J Neurochem. 1999 Dec;73(6):2286-98.; J Biol Chem. 2002 Jan 11;277(2):1619-27.; Biochem Biophys Res Commun. 2002 Jul 19;295(3):713-23.) | Transcriptional activation (J Biol Chem. 1997 Dec 26;272(52):33353-9.; J Neurochem. 1999 Dec;73(6):2286-98.; J Biol Chem. 2002 Jan 11;277(2):1619-27.) |  |
| MAGEA1 | MAGEA1 S9 | chrX:152139214-152139233 | 0.77183 | NA | EMSA, ChIP (Cancer Res. 2005 Sep 1;65(17):7751-62.) | ND |  |
| NY-ESO-1 | NY-ESO-1/CTAG1B prom. | chrX:153465958-153465977 | 0.88007 | M2 | EMSA, ChIP (Cancer Res. 2005 Sep 1;65(17):7763-74.) | ND |  |
|  | NY-ESO-1/CTAG1A prom. | chrX:153535484-153535503 | 0.88007 | M2 | EMSA, ChIP (Cancer Res. 2005 Sep 1;65(17):7763-74.) | ND |  |
| EIF2S3 | EIF2S3 site 1 | chrX:23982322-23982341 | 0.69402 | NA | EMSA, DNAse footprint, ChIP (Dev Cell. 2005 Jan;8(1):31-42.) | ND | Escape from X chromosome inactivation |
|  | EIF2S3 site 2 | chrX:23982434-23982453 | 0.83693 | M1 | EMSA, DNAse footprint, ChIP (Dev Cell. 2005 Jan;8(1):31-42.) | ND | Escape from X chromosome inactivation |
| XIST | XIST prom. | chrX:72989278-72989297 | 0.77047 | NA | EMSA, DNAse footprint, ChIP (Hum Mol Genet. 2005 Apr 1;14(7):953-65.) | Enhancer-blocking (Hum Mol Genet. 2005 Apr 1;14(7):953-65.) | X chromosome inactivation |

1. Mouse sites

| **Locus** | **Site name** | **Coordinates (UCSC July 2007)** | **PWM score** | **Class** | **Method of identification** | **Associated activity** | **Associated biological process** |
| --- | --- | --- | --- | --- | --- | --- | --- |
| Pax6 | Pax6 prom. | chr2:105507855-105507874 | 0.89451 | M2 | EMSA (J Biol Chem. 2004 Jun 25;279(26):27575-83.), ChIP (Genes Dev. 2006 Sep 1;20(17):2349-54.) | Enhancer-blocking (J Biol Chem. 2004 Jun 25;279(26):27575-83.) |  |
| p19ARF | p19ARF prom. | chr4:88546906-88546925 | 0.79794 | M1 | EMSA (Cancer Res. 2002 Jan 1;62(1):48-52.) | Transcriptional activation (Cancer Res. 2002 Jan 1;62(1):48-52.) |  |
| Irak2 | Irak2 prom. | chr6:113588429-113588448 | 0.87528 | M2 | EMSA (J Mol Biol. 2005 Feb 18;346(2):411-22.) | ND |  |
| beta-globin | 3'HS1 | chr7:110941604-110941623 | 0.88787 | M2 | EMSA (Mol Cell Biol. 2002 Jun;22(11):3820-31.), ChIP (Mol Cell Biol. 2003 Aug;23(15):5234-44; Genes Dev. 2006 Sep 1;20(17):2349-54.) | Enhancer-blocking (Mol Cell Biol. 2002 Jun;22(11):3820-31., Mol Cell Biol. 2003 Aug;23(15):5234-44), long-range interactions (Genes Dev. 2006 Sep 1;20(17):2349-54.; Nat Genet. 2003 Oct;35(2):190-4.; Mol Cell. 2002 Dec;10(6):1453-65) |  |
|  | 5'HS5 | chr7:111023154-111023173 | 0.93347 | M3 | EMSA (Mol Cell Biol. 2002 Jun;22(11):3820-31.), ChIP (Mol Cell Biol. 2003 Aug;23(15):5234-44; Genes Dev. 2006 Sep 1;20(17):2349-54.) | Enhancer-blocking (Mol Cell Biol. 2002 Jun;22(11):3820-31.), long-range interactions (Genes Dev. 2006 Sep 1;20(17):2349-54.; Nat Genet. 2003 Oct;35(2):190-4.; Mol Cell. 2002 Dec;10(6):1453-65) |  |
|  | HS-62.5 | chr7:111061690-111061709 | 0.96056 | M3 | ChIP (Mol Cell Biol. 2003 Aug;23(15):5234-44; Genes Dev. 2006 Sep 1;20(17):2349-54.) | Enhancer-blocking (Mol Cell Biol. 2003 Aug;23(15):5234-44), long-range interactions (Genes Dev. 2006 Sep 1;20(17):2349-54.; Nat Genet. 2003 Oct;35(2):190-4.; Mol Cell. 2002 Dec;10(6):1453-65) |  |
|  | HS-85.5 | chr7:111091453-111091472 | 0.96193 | M3 | ChIP (Genes Dev. 2006 Sep 1;20(17):2349-54.) | Long-range interactions (Genes Dev. 2006 Sep 1;20(17):2349-54.) |  |
| H19/L23mrp | PCT6/14 | chr7:149735932-149735951 | 0.83007 | M1 | EMSA (Hum Mol Genet. 2002 Jul 1;11(14):1627-36.) | ND |  |
|  | PCT12 | chr7:149738578-149738597 | 0.92284 | M2 | EMSA, ChIP (Hum Mol Genet. 2002 Jul 1;11(14):1627-36.) | Enhancer-blocking (Hum Mol Genet. 2002 Jul 1;11(14):1627-36.) |  |
|  | PCT4 | chr7:149742651-149742670 | 0.76051 | NA | EMSA (Hum Mol Genet. 2002 Jul 1;11(14):1627-36.) | ND |  |
| H19 | m4 | chr7:149766227-149766246 | 0.82207 | M1 | EMSA (Nature. 2000 May 25;405(6785):482-5.; Curr Biol. 2000 Jul 13;10(14):853-6.), DNAse footprint, ChIP (Curr Biol. 2000 Jul 13;10(14):853-6.) | Enhancer-blocking (Nature. 2000 May 25;405(6785):482-5.), transcriptional activation (Hum Mol Genet. 2006 Oct 1;15(19):2945-54.), long-range interactions (Science. 2006 Apr 14;312(5771):269-72.; Proc Natl Acad Sci U S A. 2006 Jul 11;103(28):10684-9.; Mol Cell Biol. 2007 May;27(9):3499-510.; Mol Cell Biol. 2008 Oct;28(20):6473-82.; Hum Mol Genet. 2008 Oct 1;17(19):3021-9.) | Imprinting |
|  | m3 | chr7:149766683-149766702 | 0.87872 | M2 | EMSA (Nature. 2000 May 25;405(6785):482-5.; Curr Biol. 2000 Jul 13;10(14):853-6.), DNAse footprint, ChIP (Curr Biol. 2000 Jul 13;10(14):853-6.) | Enhancer-blocking (Nature. 2000 May 25;405(6785):482-5.), gene activation (Hum Mol Genet. 2006 Oct 1;15(19):2945-54.), long-range interactions (Science. 2006 Apr 14;312(5771):269-72.; Proc Natl Acad Sci U S A. 2006 Jul 11;103(28):10684-9.; Mol Cell Biol. 2007 May;27(9):3499-510.; Mol Cell Biol. 2008 Oct;28(20):6473-82.; Hum Mol Genet. 2008 Oct 1;17(19):3021-9.) | Imprinting |
|  | m2 | chr7:149767706-149767725 | 0.8258 | M1 | EMSA (Nature. 2000 May 25;405(6785):482-5.; Curr Biol. 2000 Jul 13;10(14):853-6.), DNAse footprint, ChIP (Curr Biol. 2000 Jul 13;10(14):853-6.) | Enhancer-blocking (Nature. 2000 May 25;405(6785):482-5.), gene activation (Hum Mol Genet. 2006 Oct 1;15(19):2945-54.), long-range interactions (Science. 2006 Apr 14;312(5771):269-72.; Proc Natl Acad Sci U S A. 2006 Jul 11;103(28):10684-9.; Mol Cell Biol. 2007 May;27(9):3499-510.; Mol Cell Biol. 2008 Oct;28(20):6473-82.; Hum Mol Genet. 2008 Oct 1;17(19):3021-9.) | Imprinting |
| H19 | m1 | chr7:149767953-149767972 | 0.88538 | M2 | EMSA (Nature. 2000 May 25;405(6785):482-5.; Curr Biol. 2000 Jul 13;10(14):853-6.), DNAse footprint, ChIP (Curr Biol. 2000 Jul 13;10(14):853-6.) | Enhancer-blocking (Nature. 2000 May 25;405(6785):482-5.), gene activation (Hum Mol Genet. 2006 Oct 1;15(19):2945-54.), long-range interactions (Science. 2006 Apr 14;312(5771):269-72.; Proc Natl Acad Sci U S A. 2006 Jul 11;103(28):10684-9.; Mol Cell Biol. 2007 May;27(9):3499-510.; Mol Cell Biol. 2008 Oct;28(20):6473-82.; Hum Mol Genet. 2008 Oct 1;17(19):3021-9.) | Imprinting |
| KvDMR | CTS2 | chr7:150482169-150482188 | 0.71349 | NA | EMSA (Mol Cell Biol. 2007 Apr;27(7):2636-47.) | Enhancer-blocking (Mol Cell Biol. 2007 Apr;27(7):2636-47.) | Imprinting |
|  | CTS1 | chr7:150482404-150482423 | 0.81041 | M1 | EMSA (Mol Cell Biol. 2007 Apr;27(7):2636-47.) | Enhancer-blocking (Mol Cell Biol. 2007 Apr;27(7):2636-47.) | Imprinting |
| Rasgrf1 | Rasgrf1 DMD160 | chr9:89774505-89774524 | 0.83818 | M1 | EMSA (Mol Cell Biol. 2005 Dec;25(24):11184-90.) | Enhancer-blocking (Mol Cell Biol. 2005 Dec;25(24):11184-90.) | Imprinting |
|  | Rasgrf1 DMD230 | chr9:89774572-89774591 | 0.74587 | NA | EMSA (Mol Cell Biol. 2005 Dec;25(24):11184-90.) | Enhancer-blocking (Mol Cell Biol. 2005 Dec;25(24):11184-90.) | Imprinting |
|  | Rasgrf1 DMD320 | chr9:89774715-89774734 | 0.7615 | NA | EMSA (Mol Cell Biol. 2005 Dec;25(24):11184-90.) | Enhancer-blocking (Mol Cell Biol. 2005 Dec;25(24):11184-90.) | Imprinting |
| Grb10 | Grb10 DMR | chr11:11969805-11969824 | 0.74926 | NA | EMSA (Nucleic Acids Res. 2003 Mar 1;31(5):1398-406.) | ND | Imprinting |
| Th2 cytokine | CBS1 | chr11:53403936-53403955 | 0.96233 | M3 | ChIP (J Immunol. 2009 Jan 15;182(2):999-1010.) | Transcriptional activation (J Immunol. 2009 Jan 15;182(2):999-1010.) | T-cell development |
|  | CBS2 | chr11:53418551-53418570 | 0.82761 | M1 | ChIP (J Immunol. 2009 Jan 15;182(2):999-1010.) | Transcriptional activation (J Immunol. 2009 Jan 15;182(2):999-1010.) | T-cell development |
|  | CBS3 | chr11:53441578-53441597 | 0.77753 | NA | ChIP (J Immunol. 2009 Jan 15;182(2):999-1010.) | Transcriptional activation (J Immunol. 2009 Jan 15;182(2):999-1010.) | T-cell development |
|  | CBS4 | chr11:53445896-53445915 | 0.82399 | M1 | ChIP (J Immunol. 2009 Jan 15;182(2):999-1010.) | Transcriptional activation (J Immunol. 2009 Jan 15;182(2):999-1010.) | T-cell development |
|  | CBS5 | chr11:53456425-53456444 | 0.91691 | M2 | ChIP (J Immunol. 2009 Jan 15;182(2):999-1010.) | Transcriptional activation (J Immunol. 2009 Jan 15;182(2):999-1010.) | T-cell development |
| Th2 cytokine | CBS6 | chr11:53549257-53549276 | 0.83444 | M1 | ChIP (J Immunol. 2009 Jan 15;182(2):999-1010.) | Transcriptional activation (J Immunol. 2009 Jan 15;182(2):999-1010.) | T-cell development |
|  | CBS7 | chr11:53550653-53550672 | 0.8927 | M2 | ChIP (J Immunol. 2009 Jan 15;182(2):999-1010.) | Transcriptional activation (J Immunol. 2009 Jan 15;182(2):999-1010.) | T-cell development |
| Gtl2 | Gtl2 (b) | chr12:111506460-111506479 | 0.9046 | M2 | Sequence (Genome Res. 2001 Dec;11(12):2085-94.) | ND | Imprinting |
| Igh | CTS28 | chr12:114460423-114460442 | 0.90025 | M2 | EMSA, ChIP (Mol Cell Biol. 2005 Feb;25(4):1511-25.) | Enhancer-blocking (Mol Cell Biol. 2005 Feb;25(4):1511-25.) | B-cell development |
|  | CTS26 | chr12:114460688-114460707 | 0.9198 | M2 | EMSA, ChIP (Mol Cell Biol. 2005 Feb;25(4):1511-25.) | Enhancer-blocking (Mol Cell Biol. 2005 Feb;25(4):1511-25.) | B-cell development |
|  | CTS27 | chr12:114460688-114460707 | 0.9198 | M2 | EMSA, ChIP (Mol Cell Biol. 2005 Feb;25(4):1511-25.) | Enhancer-blocking (Mol Cell Biol. 2005 Feb;25(4):1511-25.) | B-cell development |
|  | CTS25 | chr12:114461160-114461179 | 0.77782 | NA | EMSA, ChIP (Mol Cell Biol. 2005 Feb;25(4):1511-25.) | Enhancer-blocking (Mol Cell Biol. 2005 Feb;25(4):1511-25.) | B-cell development |
|  | CTS20 | chr12:114462603-114462622 | 0.8339 | M1 | EMSA, ChIP (Mol Cell Biol. 2005 Feb;25(4):1511-25.) | Enhancer-blocking (Mol Cell Biol. 2005 Feb;25(4):1511-25.) | B-cell development |
|  | CTS7 | chr12:114465973-114465992 | 0.76817 | NA | EMSA (Mol Cell Biol. 2005 Feb;25(4):1511-25.) | ND | B-cell development |
|  | CTS5 | chr12:114466349-114466368 | 0.82761 | M1 | EMSA (Mol Cell Biol. 2005 Feb;25(4):1511-25.) | ND | B-cell development |
| TCRalpha | Tad1 HS1' 730-743 | chr14:54847756-54847775 | 0.90186 | M2 | EMSA, ChIP (J Biol Chem. 2004 Jun 11;279(24):25381-9.) | Enhancer-blocking (J Biol Chem. 2004 Jun 11;279(24):25381-9.) | T-cell development |
| c-myc | c-myc murine 5' boundary | chr15:64026040-64026059 | 0.96781 | M3 | ChIP (Mol Cell Biol. 2003 Dec;23(24):9338-48.) | ND |  |
|  | Murine site A (P2) | chr15:64028183-64028202 | 0.74564 | NA | ChIP (Mol Cell Biol. 2003 Dec;23(24):9338-48.) | ND |  |
| Xite | Xite 4 |  | 0.55245 | NA | EMSA, ChIP (Nat Genet. 2007 Nov;39(11):1390-6.) | Chromosome pairing (Nat Genet. 2007 Nov;39(11):1390-6.) | X chromosome inactivation |
|  | Xite 3 | chrX:100634046-100634065 | 0.74876 | NA | EMSA, DNAseI footprint, ChIP (Dev Cell. 2005 Jan;8(1):31-42.) | Chromosome pairing (Nat Genet. 2007 Nov;39(11):1390-6.) | X chromosome inactivation |
|  | Xite 2 | chrX:100635932-100635951 | 0.63647 | NA | EMSA, ChIP (Nat Genet. 2007 Nov;39(11):1390-6.) | Chromosome pairing (Nat Genet. 2007 Nov;39(11):1390-6.) | X chromosome inactivation |
|  | Xite 1 | chrX:100636344-100636363 | 0.8934 | M2 | EMSA, ChIP (Nat Genet. 2007 Nov;39(11):1390-6.) | Chromosome pairing (Nat Genet. 2007 Nov;39(11):1390-6.) | X chromosome inactivation |
| Tsix | Tsix F | chrX:100643230-100643253 | 0.81527 | M1 | ChIP (Mol Cell. 2007 Jan 12;25(1):43-56.) | ND | X chromosome inactivation |
|  | Tsix C | chrX:100643692-100643711 | 0.69967 | NA | EMSA, ChIP (Science. 2002 Jan 11;295(5553):345-7.), ChIP (Mol Cell. 2007 Jan 12;25(1):43-56.) | Enhancer-blocking (Science. 2002 Jan 11;295(5553):345-7.), | X chromosome inactivation |
|  | Tsix D (DXPas34) | chrX:100644826-100644845 | 0.71126 | NA | EMSA (Science. 2002 Jan 11;295(5553):345-7.) | Enhancer-blocking (Science. 2002 Jan 11;295(5553):345-7.), transcriptional activation (Proc Natl Acad Sci U S A. 2006 May 9;103(19):7390-5., Dev Cell. 2007 Jan;12(1):57-71., Mol Cell. 2007 Jan 12;25(1):43-56.), chromosome pairing (Nat Genet. 2007 Nov;39(11):1390-6.) | X chromosome inactivation |
|  | Tsix B | chrX:100644826-100644845 | 0.75335 | NA | EMSA (Science. 2002 Jan 11;295(5553):345-7.) | Enhancer-blocking (Science. 2002 Jan 11;295(5553):345-7.), transcriptional activation (Proc Natl Acad Sci U S A. 2006 May 9;103(19):7390-5., Dev Cell. 2007 Jan;12(1):57-71., Mol Cell. 2007 Jan 12;25(1):43-56.) | X chromosome inactivation |
|  | Tsix E | chrX:100645915-100645934 | 0.63351 | NA | Sequence (Nat Genet. 2007 Nov;39(11):1390-6.) | Chromosome pairing (Nat Genet. 2007 Nov;39(11):1390-6.) | X chromosome inactivation |
|  | Tsix A | chrX:100646707-100646726 | 0.84218 | M1 | EMSA, ChIP (Science. 2002 Jan 11;295(5553):345-7.), ChIP (Mol Cell. 2007 Jan 12;25(1):43-56.) | Enhancer-blocking (Science. 2002 Jan 11;295(5553):345-7.), chromosome pairing (Nat Genet. 2007 Nov;39(11):1390-6.) | X chromosome inactivation |
| Xist | Xist P2 | chrX:100677069-100677088 | 0.87911 | M2 | ChIP (Genes Dev. 2006 Oct 15;20(20):2787-92.) | ND | X chromosome inactivation |
|  | Xist P1 | chrX:100679567-100679586 | 0.90233 | M2 | ChIP (Hum Mol Genet. 2005 Apr 1;14(7):953-65.; Genes Dev. 2006 Oct 15;20(20):2787-92.) | ND | X chromosome inactivation |
| Jarid1c | Jarid1c Site 1 | chrX:148667472-148667491 | 0.7199 | NA | EMSA, DNaseI footprint, ChIP (Dev Cell. 2005 Jan;8(1):31-42.) | Enhancer-blocking (Dev Cell. 2005 Jan;8(1):31-42.) | Escape from X chromosome inactivation |
| Jarid1c | Jarid1c Site 2 | chrX:148667722-148667741 | 0.80766 | M1 | EMSA, DNaseI footprint, ChIP (Dev Cell. 2005 Jan;8(1):31-42.) | Enhancer-blocking (Dev Cell. 2005 Jan;8(1):31-42.) | Escape from X chromosome inactivation |
|  | Jarid1c Site 3 | chrX:148668055-148668074 | 0.77135 | NA | EMSA, ChIP (Dev Cell. 2005 Jan;8(1):31-42.) | Enhancer-blocking (Dev Cell. 2005 Jan;8(1):31-42.) | Escape from X chromosome inactivation |
| Eif2s3x | Eif2s3x Site 2 | chrX:91458568-91458587 | 0.83016 | M1 | EMSA, DNAseI footprint, ChIP (Dev Cell. 2005 Jan;8(1):31-42.) | ND | Escape from X chromosome inactivation |
|  | Eif2s3x Site 1 | chrX:91459094-91459113 | 0.89465 | M2 | EMSA, DNAseI footprint, ChIP (Dev Cell. 2005 Jan;8(1):31-42.) | ND | Escape from X chromosome inactivation |
